# Targeting the Negative Feedback of Adenosine‐A2AR Metabolic Pathway by a Tailored Nanoinhibitor for Photothermal Immunotherapy

**DOI:** 10.1002/advs.202104182

**Published:** 2022-03-20

**Authors:** Yiqiong Liu, Ying Liu, Dailin Xu, Jie Zang, Xiao Zheng, Yuge Zhao, Yan Li, Ruiqing He, Shuangrong Ruan, Haiqing Dong, Jingjing Gu, Yan Yang, Qian Cheng, Yongyong Li

**Affiliations:** ^1^ Shanghai Skin Disease Hospital The Institute for Biomedical Engineering & Nano Science School of Medicine Tongji University Shanghai 200092 China; ^2^ Institute of acoustics School of Physics Science and Engineering Tongji University Shanghai 200092 China

**Keywords:** adenosine, adenosine 2A receptor metabolic checkpoint, immunogenic cell death, negative feedback, polydopamine nanoparticles

## Abstract

The metabolite adenosine plays an important immunosuppressive role in the tumor microenvironment (TME) through its ligation with the metabolic checkpoint adenosine 2A receptor (A2AR). Here, an adenosine‐A2AR negative feedback pathway is highlighted during photothermal‐induced immunogenic cell death (ICD). Adenosine, hydrolyzed from ATP, is amplified during the photothermal‐induced ICD process. It is possible to achieve a robust ICD‐based immunotherapy via targeting the adenosine‐A2AR metabolic pathway. In this regard, an A2AR inhibitor‐loaded polydopamine nanocarrier masked by an acid‐sensitive PEG shell is designed to enable tumor‐specific delivery and photothermal‐induced ICD simultaneously. Upon reaching the acidic TME, the PEG shell selectively detaches and exposes the adhesive polydopamine layer, causing the inhibitors to accumulate at the tumor tissue. The accumulated inhibitors attenuate adenosine's metabolically suppressive effect and strengthen the ICD immune response. It occurs through promoting dendritic cell (DC) activation, increasing CD8^+^ T lymphocyte infiltration, and reducing the myeloid‐derived suppressor cell (MDSC) population. Furthermore, this synergistic therapy significantly regresses the primary tumor, inhibits distal tumor growth, and prevents lung metastasis. The study highlights a strategy to enhance the immunotherapy efficacy of ICD by blocking the metabolic checkpoint A2AR using advanced nanomaterials.

## Graphical abstract

In this study, the existence of the adenosine 2A receptor (A2AR) negative feedback pathway during photothermal therapy (PTT)‐induced immunogenic cell death (ICD) is validated. An A2AR inhibitor‐loaded polydopamine nanocarrier masked by an acid‐sensitive PEG shell is designed to simultaneously enable tumor‐specific inhibitor delivery and photothermal effect‐induced ICD. The strategy attenuates adenosine's metabolically suppressive effects and effectively strengthens the ICD immune response.

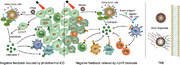

## 1 Introduction

Immune checkpoint blockers have shown exciting preclinical and clinical anticancer effects using monoclonal antibodies against cytotoxic T‐lymphocyte‐associated protein 4 and programmed cell death protein 1.^[^ ^1^ ^]^ However, their limited success in solid tumors, as well as the growing numbers of refractory patients, necessitates a deeper understanding of the molecular suppression mechanisms within the harsh tumor microenvironment (TME).^[^ ^2^ ^]^ Recently, the principal roles of immune metabolism in modulating immune responses have been recognized.^[^ ^3^ ^]^ Metabolic dysfunction commonly occurs in the TME and can severely compromise the antitumor functions of immune cells, as exemplified by the accumulation of lactate and kynurenine.^[^ ^4^ ^]^

Adenosine, an immunosuppressive metabolite, has recently gained attention as a potential target for overcoming tumor immune evasion.^[^ ^5^ ^]^ Due to the robust metabolic activity, adenosine presents in TME 17 times higher than that in normal tissue.^[^ ^6^ ^]^ As hydrolyzed from adenosine triphosphate (ATP), adenosine binds the adenosine 2A receptor (A2AR) on immune cells and cancer cells to exert pleiotropic immunosuppressive effects.^[^ ^7^ ^]^ This binding enables tumor cells to escape immune surveillance by limiting the functionality of multiple infiltrating protective immune cells, including T cells,^[^ ^8^ ^]^ DCs,^[^ ^9^ ^]^ and natural killer (NK) cells.^[^ ^10^ ^]^ The adenosine‐A2AR pathway also enhances the activity of immunosuppressive cell types, such as MDSCs and regulatory T cells (Tregs), and promotes survival, proliferation, migration, and invasion of tumor cells.^[^ ^10^, ^11^ ^]^ In order to relieve adenosine‐A2AR pathway immunosuppression, A2AR blockade has been found to increase NK cell maturation in the TME,^[^ ^12^ ^]^ improving DC cross‐presentation function,^[^ ^13^ ^]^ and reduce tumor aggregation of Tregs and MDSCs.^[^ ^14^ ^]^ A phase I clinical trial with a small‐molecule A2AR antagonist for the treatment of refractory renal cancer displayed durable clinical benefits.^[^ ^15^ ^]^

Various combination strategies for mitigating immunosuppression during antitumor immune responses, especially via immunogenic cell death (ICD),^[^ ^16^ ^]^ have been developed, including photothermal therapy (PTT)‐induced ICD,^[^ ^17^ ^]^ photodynamic therapy (PDT)‐induced ICD,^[^ ^18^ ^]^ and chemotherapy‐induced ICD.^[^ ^19^ ^]^ ICD is a cell death mode that induces tumor immunogenicity, releases antigens, and upregulates “eat me” and “danger” signals, such as high mobility group box 1 (HMGB1), calreticulin (CRT), and ATP.^[^ ^20^ ^]^ This in situ vaccine effect can potentiate antitumor immune responses by promoting antigen‐presenting cell (APC) activation and triggering antigen‐specific CD8^+^ T‐cell responses.^[^ ^21^ ^]^ However, the previous report suggested that ICD effects might be insufficient to cause robust antitumor immunity.^[^ ^22^ ^]^ It means that negative feedback mechanisms exist, as in antitumor immunotherapy. Considering that a significant increase in ATP is an essential feature of ICD, we hypothesized that the adenosine‐A2AR pathway plays a crucial immunosuppressive regulatory role in ICD.

**Figure 1:**
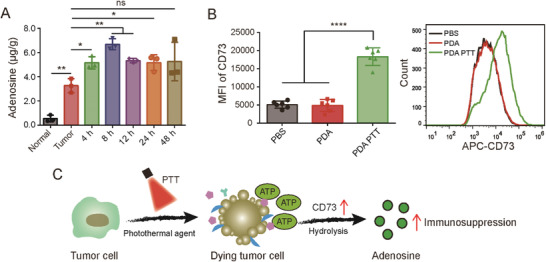
A) Tumor adenosine level changes after PTT treatment (48 °C, 10 min) as detected by high‐performance liquid chromatography (HPLC). Normal: normal tissue; Tumor: tumor without treatment; n = 3. B) CD73 expression before and after PTT treatment in vivo detected by flow cytometry, n = 6. C) Schematic illustration of the negative feedback of adenosine‐A2AR metabolic pathway in PTT‐induced ICD. Data are expressed as the means ± SD. Statistical significance is defined as *p < 0.05, **p < 0.01, ***p < 0.001, ****p < 0.0001 and ns represents no significant difference.

**Scheme 1:**
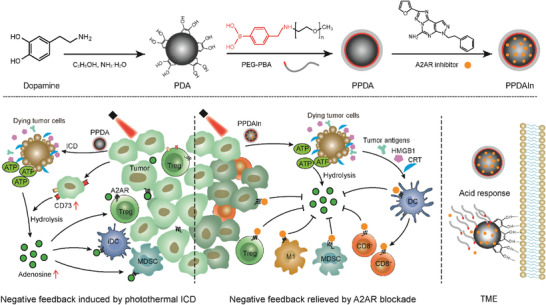
A strategy for enhancing ICD immunotherapy efficacy by blocking metabolic checkpoint A2AR using TME‐responsive PPDAIn (PPDA loaded with inhibitor SCH58261) NPs. M1, M1‐type macrophages. iDC, immature dendritic cells.

In this study, we found that PTT treatment led to a significant upregulation of adenosine in tumor tissues (**Figure** **1**), suggesting that the adenosine‐A2AR pathway plays a counterbalancing role. On this basis, we developed an A2AR inhibitor‐loaded polydopamine nanocarrier masked by an acid‐sensitive PEG shell to enable tumor‐specific delivery and PTT‐enhanced ICD immunotherapy simultaneously. The selected polydopamine (PDA) nanoparticle (NP), a biocompatible and biomimetic nanomaterial,^[^ ^23^ ^]^ acts as a photothermal ICD inducer and nanocarrier for A2AR inhibitor SCH58261. Most small‐molecule A2AR antagonists display poor water‐solubility and targeting, presenting a major challenge to their therapeutic delivery and preclinical evaluation.^[^ ^24^ ^]^ To increase tumor accumulation of A2AR antagonists, we designed an acid‐responsive detachable PEG shell outside the PDA (PPDA). Upon reaching the acidic tumor milieu, the PEG shell is released to present the inhibitor‐loaded PDA, whose mussel‐mimicking adhesive properties anchor it to the tumor tissue, leading to tumor retention and accumulation. The blockade of the metabolic checkpoint A2AR decreased the metabolic stress of adenosine in tumor‐infiltrating immune cells and enhanced ICD‐mediated effective antitumor immune responses (**Scheme** **1**). The strategy provides new insights into improving ICD immunotherapy by counterbalancing the negative feedback of adenosine.

## 2 Results and Discussion

### 2.1 Negative Feedback Regulation During ICD

To determine if the negative feedback of adenosine‐A2AR regulation occurs during ICD, we subjected 4T1 tumor‐bearing mice to PTT. PDA, not inherently affecting the adenosine excretion (Figure S1, Supporting Information), was chosen as the photothermal agent. Tumor adenosine content was markedly upregulated after PTT, reaching a peak at 8 h that was twofold higher than that in non‐treated tumors (Figure 1A), suggesting that the immunosuppressive metabolite adenosine was produced in response to ICD. In addition, the adenosine concentration was significantly higher in tumor tissue compared to normal tissue. Furthermore, we found that CD73 expression, which is necessary for ATP hydrolysis, was also markedly upregulated in tumor cells after PTT (Figure 1B). These results suggested that the negative feedback regulation was related to the immunosuppressive metabolite adenosine during ICD (Figure 1C).

### 2.2 Preparation and Characterization of PPDA

**Figure 2:**
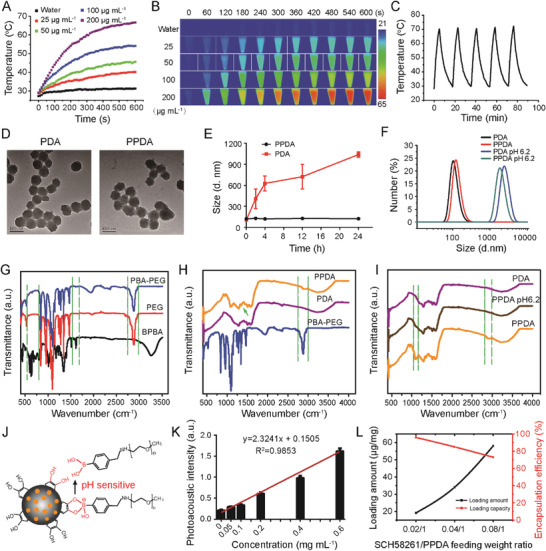
A) Temperature profiles of PDA NPs at various concentrations after 808 nm laser (0.5 W cm^−2^) irradiation. B) Infrared thermal images of water and PDA NPs irradiated for 600 s (0.5 W cm^−2^). C) Temperature changes of PDA NPs (200 µg mL^−1^) in five consecutive cycles of photothermal irradiation (808 nm, 0.5 W cm^−2^) and natural cooling. D) TEM images of PDA and PPDA (scale bar: 100 nm). E) Size changes of PDA and PPDA NPs in PBS (pH = 7.4) in 24 h, n = 3. F) Size variations of PDA and PPDA after acidic PBS (pH = 6.2) treatment for 3.5 h. G) FTIR spectra of BPBA, PEG, and PBA‐PEG. H) FTIR spectra of PBA‐PEG, PDA, and PPDA, and I) shell shedding of PPDA after acid treatment. J) Schematic illustration of the pH‐responsive PBA‐PEG shell shedding. K) The PA signal produced by PPDA at different concentrations was linearly dependent on its concentration (R^2^ = 0.985). L) Loading amount and encapsulation efficiency of A2AR antagonist SCH58261.

We synthesized photosensitive PDA NPs using a previously reported method.^[^ ^25^ ^]^ To assess PDA's photothermal efficiency, we subjected different concentrations of PDA to irradiation by an 808 nm laser with a power density of 0.5 W cm^−2^. The temperature of all samples increased with irradiation time and correlated with increasing concentration. The temperature of PDA (200 µg mL^−1^) quickly increased up to 48 °C at t = 130 s and 66 °C at t = 550 s. In contrast, pure water (a negative control) showed no significant temperature increase under the same conditions (**Figure** **2**A). Infrared thermal images further demonstrated the excellent photothermal conversion efficiency of PDA (Figure 2B). In addition, Figure S2, Supporting Information, showed an evident temperature increase dependent on laser power density. The ΔT of PDA remained nearly unchanged after five ON/OFF cycles of irradiation (Figure 2C), indicating tremendous photothermal stability.

We then characterized PPDA synthesis and its acid responsiveness. To endow PDA with an acid‐responsive detachable PEG shell, we first prepared 4‐bromomethyl phenylboronic acid (BPBA) linked amino polyethylene glycol (PBA‐PEG). PPDA was obtained by tethering PBA‐PEG to PDA via conjugation of the boronic acid and catechol groups. Scanning electronic microscopy (SEM) and transmission electron microscopy (TEM) showed a homogeneous spherical morphology and uniform size for both PDA and PPDA (Figure 2D, Figure S3, Supporting Information). Compared with PDA, PPDA had a slightly increased particle size (≈10 nm) with surface charge converting from −37 mV to approximately −4 mV (Figure S4, Supporting Information). Moreover, compared to PDA, PPDA exhibited excellent stability in phosphate‐buffered saline (PBS, pH 7.4) as monitored by a dynamic light scattering (DLS) assay. The size of PDA increased to ≈400 nm after a 2‐h suspension in PBS and to ≈1000 nm after 24 h (Figure 2E, Figure S5, Supporting Information). In contrast, PPDA had a narrow size distribution and minor changes in size under the same conditions, which is a crucial feature for maintaining nanocarrier homogeneity, suggesting the successful modification of PBA‐PEG.

To further characterize PBA‐PEG and PPDA, we measured the characteristic chemical peaks using Fourier transform infrared spectroscopy (FTIR) and ^1^H NMR spectroscopy. The FTIR spectra showed that PBA‐PEG had a broadened absorbance in 600 to 780 cm^−1^, which is associated with the benzene ring of phenylboronic acid, a peak around 1600 cm^−1^, which is related to C═C stretching vibration, and a peak around 2890 cm^−1^, which correlates with —CH_2_— symmetrical vibration, indicating successful synthesis of PBA‐PEG (Figure 2G). After PBA‐PEG modification, the PPDA FTIR spectra exhibited typical —CH_2_— symmetrical vibration (2900 cm^−1^) and B—O stretching vibration (1346 cm^−1^), indicative of the successful PPDA construction (Figure 2H). Complementary to FTIR, ^1^H NMR spectroscopy provided additional evidence that PBA‐PEG had effectively grafted onto PDA (Figures S6 and S7, Supporting Information). To validate whether PPDA was responsive to acid, we dispersed PPDA in PBS (pH 6.2). DLS showed significant aggregation of both PPDA and PDA (Figure 2F). The PPDA aggregation can be attributed to the broken pH‐sensitive bond between PBA‐PEG and PDA, leading to detachment of the PBA‐PEG shell from PDA (Figure 2J) and subsequent PDA aggregation. To validate this, we treated PPDA with an acid solution, subjected it to ultrafiltration to remove the detached PBA‐PEG (the obtained NP denoted as aPDA), and then lyophilized it for FTIR testing. The characteristic PBA‐PEG peaks were absent instead of the appearance of peaks similar to PDA (Figure 2I), supporting PPDA's acid responsiveness. The amount of PBA‐PEG conjugated onto PDA was analyzed by detaching PBA‐PEG from PDA upon acid treatment, which showed 0.204 ± 0.010 mg of PBA‐PEG modified onto 1.0 mg of PDA NPs.

Next, we loaded the A2AR antagonist SCH58261 at a therapeutically relevant dose (1 mg kg^−1^) into PPDA via noncovalent interactions,^[^ ^10^, ^26^ ^]^ such as *π*‐*π* stacking, hydrogen bonding, and hydrophobic interactions, with a high loading efficiency (Figure 2L). SCH58261 exhibited an irradiation‐responsive release profile, whereas weak acid condition (pH 6.2) only had a slightly accelerated release (Figure S8, Supporting Information). The accumulative release of SCH58261 upon light irradiation in pH 6.2 reached 80.0% at the time point of 24 h, nearly twofold higher than the group without irradiation (41.3%).

The photothermal conversion efficiency of PDA and PPDA was calculated to be 46.0% and 44.8%, respectively. Next, to investigate the capacity of PPDA as photoacoustic imaging (PAI) contrast agent, we measured the photoacoustic (PA) signals of PPDA aqueous solutions at different concentrations. The PA signals increased linearly with PPDA concentration (R^2^ = 0.9853) (Figure 2K), suggesting that in vivo PPDA accumulation could be measured quantitatively using PAI.

### 2.3 Investigation of PPDA Adhesive Properties and Tumor Accumulation

**Figure 3:**
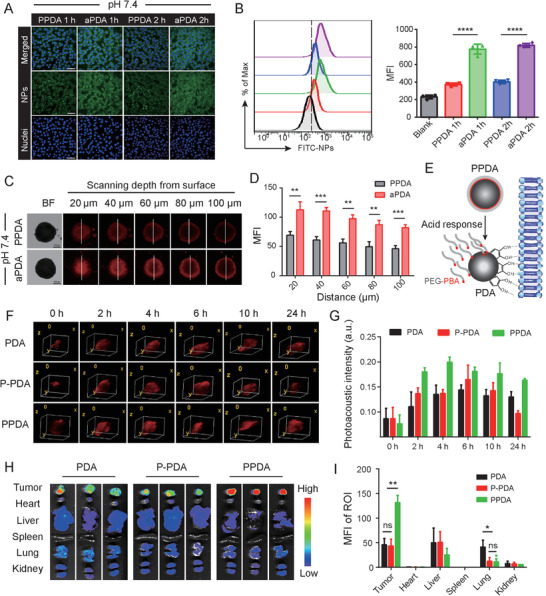
A) CLSM images of 4T1 cells after incubation with FITC‐PPDA or FITC‐aPDA (scale bar: 50 µm). B) Cellular mean fluorescence intensity (MFI) quantitatively analyzed by flow cytometry, n = 3. C) CLSM Z‐stack images of 4T1 tumor spheroids after treatment with PPDA or aPDA for 12 h. Scale bar: 250 µm. D) Quantitative analysis of fluorescence intensity in panel (C) images by Image J, n = 3. E) Schematic illustration of PPDA adhesion mechanism. F) PAI of 4T1 tumor‐bearing mice after tail‐vein injection of PDA, P‐PDA, or PPDA, n = 4. G) Average tumor PA intensity measured at different time points after intravenous injection of NPs, n = 4. H) Ex vivo fluorescence images and I) quantitative analysis of fluorescence signals from major organs and tumors 24 h post‐injection, n = 3. Data are expressed as the means ± SD. Statistical significance is defined as *p < 0.05, **p < 0.01, ***p < 0.001, ****p < 0.0001 and ns represents no significant difference.

Here, we tested whether the acid‐responsive PPDA had robust tumor cell adhesion. We assessed the biocompatibility of PPDA using a cell counting kit‐8 (CCK8) assay. Even at the highest tested dose (1.0 mg mL^−1^), PPDA had neglectable cytotoxicity, indicative of excellent biosafety (Figure S9, Supporting Information). To visualize the interaction of PPDA with tumor cells, we incubated FITC‐labeled PPDA or aPDA with 4T1 cells in a medium with pH 7.4 for 1 or 2 h. The aPDA group displayed significantly stronger cellular fluorescence than PPDA at 1 h, as well as more background fluorescence, suggesting PDA adhered to broad surface types, including cells and Petri dishes (**Figure** **3**A, Figure S10, Supporting Information). Similar results were also observed at 2 h. The fluorescence intensity of NP‐incubated 4T1 cells was further quantified using flow cytometry. The aPDA group showed a twofold higher fluorescence intensity than the PPDA group at 1 and 2 h (Figure 3B). The adhesive properties were also tested using a tumor spheroid model. 4T1 tumor spheroids, simulating solid biological tumors, were cultured and used as an in vitro model. Tumor spheroids were treated with rhodamine B‐conjugated NPs for 12 h. Confocal laser scanning microscope (CLSM) Z‐stack scanning was then used to monitor the accumulation capability (Figure 3C,D). We detected increasing fluorescent signals after aPDA treatment compared with the PPDA treatment (Figure 3D). And fluorescence intensity profiles of the tumor cell spheroid sections by Z‐stacking scanning at different depths from the surface (20, 40, 60, 80, and 100 µm), as shown by a white line in Figure 3C, intuitively showed fluorescence distribution inside the spheroid (Figure S11, Supporting Information). The adhesive properties of PDA are due to its catechol and amine groups that play a central role in its mussel‐mimicking versatility (Figure 3E).^[^ ^27^ ^]^ The fluorescence spectra for the FITC and rhodamine‐labeled NPs showed in Figure S12, Supporting Information.

We subsequently investigated NP accumulation in tumors in vivo using PAI and fluorescence imaging. The in vivo photoacoustic sensitivity of PPDA was examined after intratumor injection at a concentration of 25 µg mL^−1^ (Figure S13, Supporting Information). The PA signal near the site of injection significantly increased during the injection process (Figure S13B–D, Supporting Information), indicating that PPDA can be detected in vivo by PAI. Additionally, to make the experimental design more rigorous, we synthesized a non‐responsive nanocarrier with a non‐shedding PEG shell (P‐PDA) as a control, characterized by FTIR (Figure S14, Supporting Information). 4T1 tumor‐bearing mice were then randomly divided into three groups and intravenously injected with PDA, PPDA, or P‐PDA for real‐time PAI assessment. Substantial intratumor accumulation was observed at 2–24 h (Figure 3F,G). The PPDA group accumulation was superior to the other two groups at all time points, likely due to its longer circulation time and its restored adhesive properties after PEG shell detachment in an acidic tumor environment. Moreover, P‐PDA was more likely to be eliminated by tumors than PDA or PPDA, suggesting that PDA adhesion enhanced tumor retention. Strikingly, tumors turned visibly black 6 h after injection of PDA, PPDA, or P‐PDA NPs, which faded gradually after 10 h (Figure S15, Supporting Information).

We used ex vivo imaging of excised tumors and organs to investigate the distribution profile of NPs in the body. Mice were euthanized 24 h after NP injection, and fluorescence signals were captured (Figure 3H). The fluorescence intensity in PPDA treated tumors was 2.88‐ and 3.04‐fold higher than that of the PDA and P‐PDA groups, respectively (Figure 3I). Fluorescence signals were barely detected in the heart and spleen. Notably, there was more PDA accumulation in the lungs compared to PPDA and P‐PDA (Figure 3I), which may be due to the adhesive properties of PDA without the protection of the PEG shell. Taken together, we successfully synthesized a pH‐sensitive PPDA nanocarrier with high tumor accumulation. The specific tumor targeting was crucial for inhibitor delivery and PTT‐induced ICD.

### 2.4 Evaluation of PTT‐Mediated ICD Induction and Changes in Adenosine Concentration

**Figure 4:**
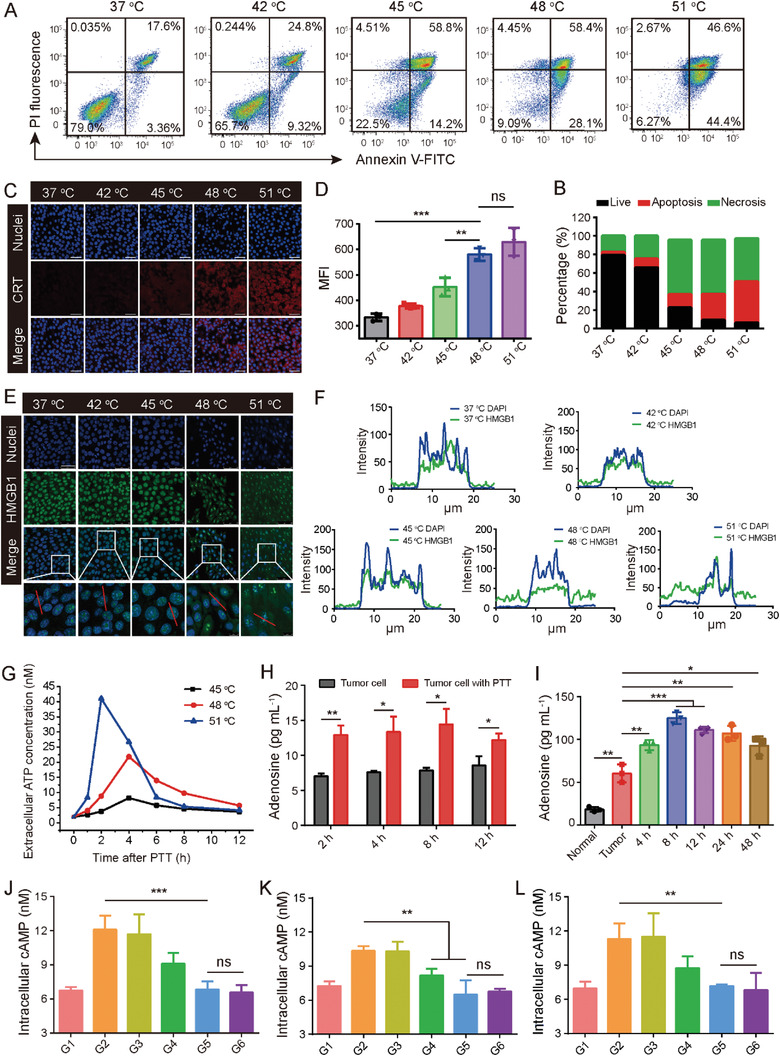
A) Flow cytometry determination of 4T1 cell apoptosis/necrosis 12 h after treatment. B) Statistical analysis of (A). C) CLSM detection of CRT exposure in 4T1 cell samples after treatment with different laser temperatures. Scale bar: 50 µm. D) Flow cytometry analysis of CRT, n = 3. E) CLSM images showing HMGB1 release from nucleus (red line indicates colocalization analysis position). Scale bar: 50 µm. F) Colocalization analysis of images in (E). G) ATP secretion profiles from 4T1 cells after treatment with different temperatures. H) Adenosine levels in 4T1‐cell culture medium after irradiation at 48 °C, n = 3. I) ELISA detection of tumor adenosine levels after PTT treatment. Normal: normal tissue; Tumor: control tumor without treatment (n = 3). The expression level of intracellular cAMP on J) DCs, K) macrophages, and L) T cells after indicated treatments. Data are expressed as the means ± SD. Statistical significance is defined as *p < 0.05, **p < 0.01, ***p < 0.001 and ns represents no significant difference.

PTT is a promising, minimally invasive therapy for various malignant tumors.^[^ ^28^ ^]^ To determine the efficacy of PPDA in PPT‐mediated tumor cell growth inhibition, we evaluated photothermal cytotoxicity using a CCK8 assay, live and dead cell staining, and an apoptotic necrosis detection assay. Upon treatment with 808 nm laser irradiation, PPDA converted light into heat energy, increasing the culture media temperature around the tumor cells. Cell viability markedly decreased when the temperature was over 45 °C for 10 min (Figure S16, Supporting Information). However, tumor cells treated with either lower temperature or PPDA alone (without irradiation) showed negligible cell death. Tumor cells treated with different temperatures were also stained with calcein‐AM and propidium iodide (PI). An obvious difference in the distribution of live cells (green) and dead cells (red) among groups was observed (Figure S17, Supporting Information). At 45 °C, cellular morphology changed, and apoptosis increased compared to treatment at 37 or 42 °C. Interestingly, tumor cells simultaneously displayed both green and red fluorescence after exposure to 48 or 51 °C. This paradoxical phenomenon occurred likely because after photothermal treatment for 4 h, although the cell membrane was destroyed, part of the esterase activity was retained. Therefore, PI could penetrate the membrane and bind to DNA, leading to red fluorescence, while calcein‐AM could interact with esterase, leading to green fluorescence. Additionally, an apoptosis assay showed a sharp decrease in the number of living cells and a considerable increase in apoptotic cells as the temperature increased (**Figure** **4**A,B). Together, these data demonstrated that PPDA effectively killed tumor cells through a near‐infrared (NIR) light irradiation‐induced photothermal effect.

PTT‐induced ICD of tumor cells is considered an effective method to achieve cytotoxicity and elicit immunological enhancement.^[^ ^29^ ^]^ ICD is characterized by releasing damage‐associated molecular pattern (DAMP) immunogenic signals, including CRT, HMGB1, and ATP, which facilitate immune responses. Here, we systematically measured DAMPs to assess PTT‐induced ICD. CRT exposure was first investigated by CLSM and flow cytometry. 4T1 cells were incubated with PPDA, and the temperature was carefully controlled at 42, 45, 48, or 51 °C by adjusting the laser power density. After staining with Percp‐CRT antibody, tumor cells showed temperature‐dependent CRT exposure. Bright CRT indicator signal (red fluorescence) was detected after irradiation at 48 and 51 °C, while nearly no fluorescence was seen in the 42 and 37 °C groups (Figure 4C). Consistent results were obtained by flow cytometry (Figure 4D). The HMGB1 release was then observed by CLSM. As shown in Figure 4E, the HMGB1 signal (green fluorescence) overlapped with the nuclear signal (blue fluorescence) in the 37, 42, and 45 °C groups. In contrast, HMGB1 had markedly diffused out of the nuclei upon 48 and 51 °C treatment, suggesting that higher PTT temperature promotes HMGB1 release. Colocalization analysis further supported these results (Figure 4F). Next, ATP secretion was monitored by a luciferase assay kit (Figure 4G). After 4T1 cells were irradiated at 51 °C, the ATP supernatant level increased 21.3 times from 0 to 2 h and then decreased rapidly over the next four hours. In the samples irradiated at 48 °C, the ATP secretion increased 10.9‐fold by 4 h and then gradually declined. Minor ATP secretion was detected in the samples irradiated at 45 °C. Collectively, these results indicated that PTT at 48 and 51 °C could effectively cause ICD.

The ATP secreted by dying tumor cells acts as a “find‐me” signal and is crucial for the immune response. However, ATP can be converted to adenosine in seconds, inducing immune suppression through the adenosine‐A2AR metabolic pathway, triggering a negative feedback mechanism.^[^ ^30^ ^]^ Thus, we quantified adenosine content in tumor cell supernatant after laser treatment using an enzyme‐linked immunosorbent assay (ELISA). The above results suggested that both 48 and 51 °C could successfully elicit ICD; however, because the higher temperature may cause damage to normal tissues, 48 °C was implemented in the following experiments. As expected, there was a markedly higher adenosine concentration at 2–12 h after PTT compared with the control (non‐treated) tumor cell group (Figure 4H). The in vivo tumor adenosine content after PTT was further monitored by ELISA. We found that adenosine concentration was markedly upregulated by 2.1‐fold 8 h after PTT compared to control tumor samples (Figure 4I). A similar trend was observed in adenosine levels using HPLC (Figure 1A). The above results suggested that PTT treatment could trigger temperature‐dependent ICD phenotype and upregulate the immunosuppressive molecule adenosine.

The profound immunosuppression of adenosine is associated with the downstream signaling molecule cyclic AMP (cAMP) after binding with A2AR. Thus, the nanoinhibitor PPDAIn (PPDA loaded with SCH58261)‐mediated cAMP inhibition was further investigated on DCs, macrophages, and T cells. The immunocytes were treated with G1) PBS, G2) adenosine, G3) adenosine plus PPDA, G4) adenosine plus nonphotoirradiated PPDAIn, G5) adenosine plus photoirradiated PPDAIn, and G6) adenosine plus free SCH58261. As shown in Figure 4J,K,L, adenosine caused obviously higher cAMP production in immunocytes compared with the PBS‐treated group. Notably, photoirradiated PPDAIn (G5) showed comparable inhibition with that of free inhibitor (G6). Photoirradiated PPDAIn significantly suppressed intracellular cAMP by 1.77, 1.59, and 1.58‐fold in DC, macrophages, and T cells, respectively, suggesting the nanoinhibitor could effectively counterbalance immunosuppression. The nonirradiated PPDAIn (G4) also decreased the intracellular cAMP. The nanocarrier PPDA alone (G3) had an unnoticeable effect on the immunocytes.

### 2.5 Immunoactivation of A2AR Nanoinhibitor on Photothermal Immune Responses In Vitro

**Figure 5:**
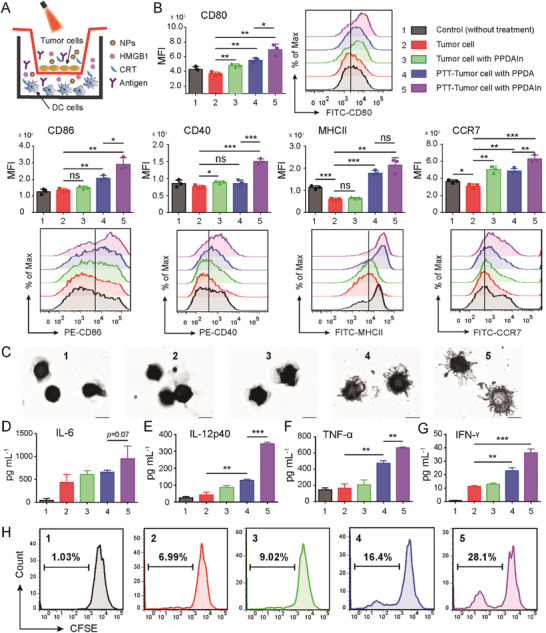
A) Schematic illustration of PTT‐induced ICD eliciting DC maturation. B) Flow cytometry analysis of BMDC maturation after 24 h culture with different tumor cell groups. BMDCs cultured without tumor cells served as a control. The histograms and corresponding peak figures represent CD80, CD86, CD40, MHCII, and CCR7 expression (gating on CD11c^+^ cells). C) BMDC morphology was imaged using an optical microscope (scale bar: 10 µm). Quantification of D) IL‐6, E) IL‐12p40, and F) TNF‐*α* secretion in BMDC suspensions, n = 3. G) Quantification of IFN‐*γ* secretion from T cell suspensions. H) The proliferation of CD3^+^ T cells harvested from spleen after coincubation with differently treated tumor cells measured using flow cytometry. T cells without any treatment served as a control (group 1). Data are expressed as the means ± SD, n = 3. Statistical significance is defined as *p < 0.05, **p < 0.01, ***p < 0.001 and ns represents no significant difference.

We measured the “vaccine” effect of PTT‐induced ICD on DCs maturation and T cell activation, and explored whether SCH58261, the adenosine receptor inhibitor, could strengthen the immune response in vitro. 4T1 tumor cells were treated with PPDA or PPDAIn with or without laser irradiation. Then, the differently treated 4T1 cells were placed into the upper layer of a Transwell and co‐cultured with bone marrow dendritic cells (BMDCs) in the lower chamber for 24 h (**Figure** **5**A). Experimental groups included BMDCs cultured with non‐treated tumor cells (2), PPDAIn treated tumor cells (3), PPDA pretreated tumor cells with PTT (4), and PPDAIn pretreated tumor cells with PTT (5). BMDCs cultured without tumor cells served as a control (1).

CD80, CD86, CD40, MHCII, and CCR7 were chosen as the specific biomarkers to determine the maturation of BMDCs by flow cytometry. The expression levels of CD80, CD86, MHCII, and CCR7 in the PPDA pretreated PTT‐tumor cells were increased by 1.50‐fold, 1.54‐fold, 3.00‐fold, and 1.47‐fold, respectively, compared to non‐treated tumor cells (Figure 5B), suggesting that PPT‐elicited ICD could induce an immune response. Remarkably, the PPDAIn pretreated PTT‐tumor cell group exhibited significantly elevated CD80, CD86, CD40, and CCR7 expression in BMDCs compared to the PPDA pretreated PTT‐tumor cell group by 1.26‐fold, 1.41‐fold, 1.76‐fold, and 1.29‐fold, respectively, indicative of enhanced BMDC maturation after A2AR blockade compared to PTT alone. The mature morphology of each group of BMDCs was imaged under the microscope (Figure 5C). The secretion of DC activation indicators including interleukin 12 (IL‐12p40), interleukin 6 (IL‐6), and tumor necrosis factor *α* (TNF‐*α*) were also evaluated by ELISA. IL‐6 was upregulated in the PPDAIn pretreated PTT‐tumor cell group (G5) compared to PPDA pretreated PTT‐tumor cells (G4) (Figure 5D). The secretion of IL‐12p40 and TNF‐*α* from BMDCs was notably higher in the PPDAIn pretreated PTT‐tumor cell group than other groups (Figure 5E,F). The results indicated that ICD combined with A2AR blockade could induce strong antitumor immunity responses.

We further assessed T‐cell proliferation to determine whether A2AR blockade influenced T cell immune responses. T cells were stained with a carboxyfluorescein (CFSE) solution, and cell proliferation was measured using flow cytometry. The experimental groups were set as that BMDC experiment. As shown in Figure 5H, PTT‐treated tumor cells stimulated T cell proliferation. Moreover, the combination of PTT with the A2AR inhibitor further increased T cell proliferation. The interferon‐*γ* (IFN‐*γ*) secretion levels were consistent with this trend (Figure 5G). These data suggested that the released adenosine could neutralize DAMP stimulatory activity, and the NP delivered A2AR inhibitor could efficiently block the immunosuppressive adenosine‐A2AR pathway and enhance PTT‐induced ICD antitumor immune responses.

### 2.6 A2AR Nanoinhibitor Enhanced Photothermal Immunotherapy Efficacy In Vivo

**Figure 6:**
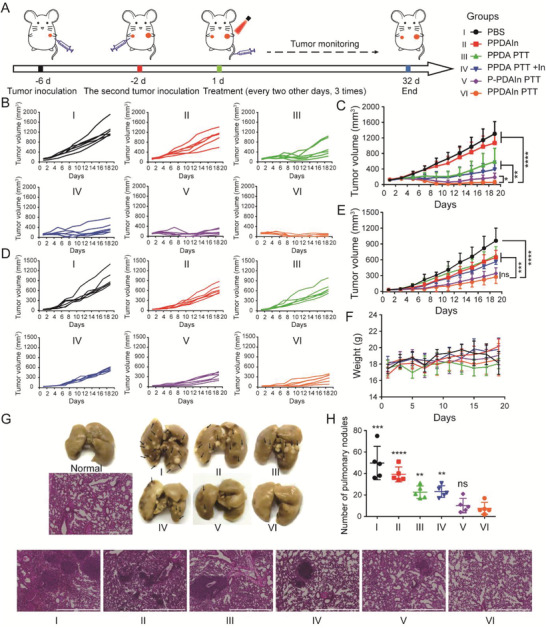
A) Schematic illustration of the in vivo experimental design. B) Primary tumor growth curves from individual 4T1 tumor‐bearing BALB/c mice in different treatment groups. C) Primary tumor growth curves with mean tumor volumes from 4T1 tumor‐bearing BALB/c mice (n = 6 biologically independent mice). The D) individual and E) averaged 4T1 tumor growth curves of distant tumors after different treatments (n = 6 biologically independent mice). F) Body weights of 4T1 tumor mice treated with different formulations (n = 6 biologically independent mice). G) Representative images of H&E stained lungs from different groups showing tumor metastasis. The black arrows refer to tumor nodules. Scale bar: 1000 µm. H) Number of pulmonary nodules after different treatments. Data are statistically compared to the PPDAIn PTT treatment group (n = 5 biologically independent mice). Data are expressed as the means ± SD. Statistical significance is defined as *p < 0.05, **p < 0.01, ***p < 0.001, ****p < 0.0001 and ns represents no significant difference.

To validate whether the proposed combination of PTT‐induced ICD and metabolic checkpoint blockade could promote antitumor effects in vivo, we established an orthotopic breast cancer model using 4T1 tumor cells. 4T1 cells were inoculated subcutaneously into the right flank of female BALB/c mice at the second mammary gland. Additionally, to investigate whether the combination therapy could exert a robust systemic immune response and inhibit an untreated distant tumor, we planted a second tumor in the contralateral side on day ‐2. Only the right flank tumor was treated with PTT on day 1, day 4, and day 7 (**Figure** **6**A). The PTT groups were irradiated by the 808 nm laser for 10 min at 48 °C.

The tumor growth curve of each mouse was monitored (Figure 6B). As shown in Figure 6C, the PPDAIn treatment moderately inhibited tumor growth compared to the PBS group, supporting the antitumor efficacy of the A2AR inhibitor. PPDA PTT‐treated mice showed delayed tumor growth, suggesting that pure PTT therapy had non‐ideal efficacy, despite being able to induce an ICD response. In contrast, mice receiving PPDAIn PTT treatment exhibited notable tumor inhibition and even tumor ablation, supporting a remarkable synergy between PTT and the A2AR inhibitor. Furthermore, PPDAIn PTT treatment was superior in inhibiting tumor growth than the non‐acid responsive delivery formula (P‐PDAIn PTT), likely due to the improved NP accumulation in tumors, resulting in more effective photothermal treatment and enriched A2AR inhibitor.

We also evaluated the untreated distant tumor growth (Figure 6D,E). The PBS‐treated group showed rapid growth. The PPDAIn, PPDA PTT, and PPDA PTT + In (inhibitor) treatments weakly influenced distant tumor growth. In contrast, P‐PDAIn PTT and PPDAIn PTT treatments, which showed robust immune response stimulation, displayed a greatly improved abscopal effect, markedly suppressing distant tumor growth and demonstrating the significant advantage of this combination therapy. We evaluated tumor cell apoptosis and necrosis of both right and left 4T1 tumors by hematoxylin and eosin (H&E) staining, which further supported the superior therapeutic effect of PPDAIn PTT treatment (Figure S18, Supporting Information). In addition, no significant weight changes were observed in the mice after treatment with different NPs, reflecting their excellent biocompatibility (Figure 6F).

The majority of cancer deaths are due to metastases, which are rarely cured effectively with traditional therapies such as surgery, chemotherapy, and radiotherapy.^[^ ^31^ ^]^ Thus, we wondered whether the combination therapy of PTT‐induced ICD and A2AR blockade could maximize the antitumor immune response and inhibit tumor metastasis. Mice were euthanized on day 32, and lungs were collected. Lungs were stained with Bouin's solution, pulmonary nodules were calculated, and lung sections were analyzed using H&E (Figure 6G,H). There were large numbers of aggressive lung metastases in the PBS‐treated group. Encouragingly, we found that the metastatic lung nodules from the PPDAIn PTT treated group were reduced compared to the other treatment groups. H&E staining further demonstrated a significant decrease in the number and invasion area of metastatic tumors in the PPDAIn PTT treated group. These results showed that PPDAIn PTT treatment could effectively destroy the local tumor, suppress a distal tumor, and prevent metastasis.

Further, histopathological assays were conducted to examine the in vivo treatment toxicity. We observed the major organs through H&E staining. There was no appreciable inflammatory infiltrate or pathological damage in any organs after any of the various treatments, indicating that PPDAIn exhibited good biocompatibility with or without NIR laser irradiation (Figure S19, Supporting Information). Moreover, the blood was collected to perform a serum biochemistry assay. Liver and kidney function marker levels were all within the normal range (Figure S20, Supporting Information), indicating that PPDAIn‐based combinational therapy can be a safe, nontoxic strategy for cancer treatment.

### 2.7 The Antitumor Immune Mechanism of A2AR Nanoinhibitor During PTT‐Induced ICD

**Figure 7:**
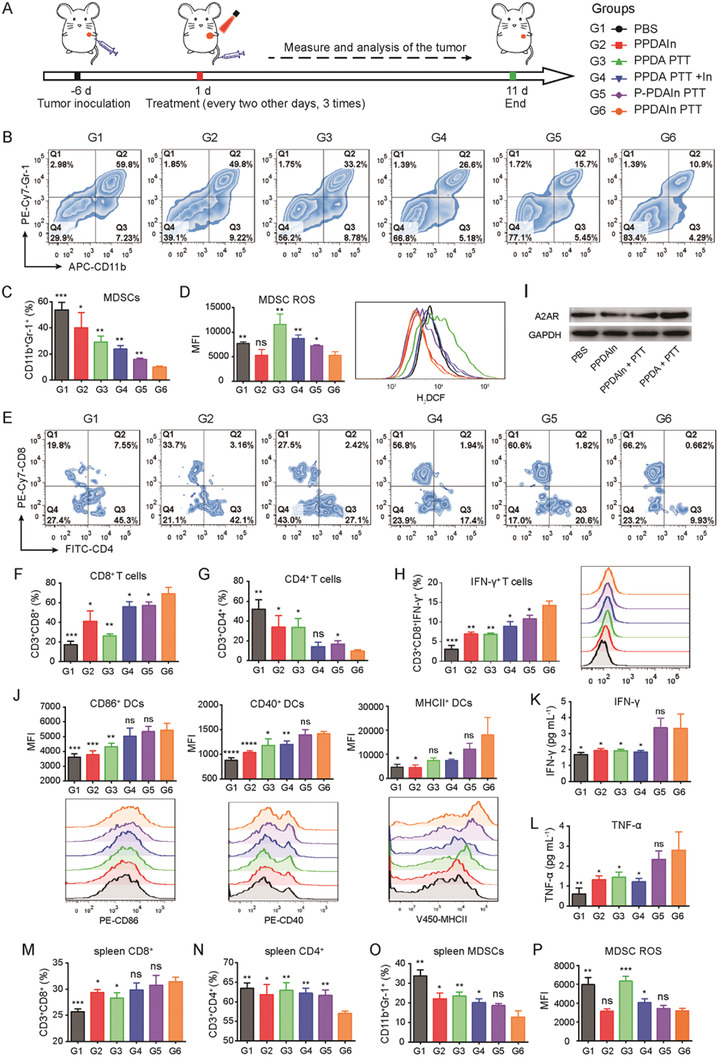
A) Schematic illustration of the in vivo experimental design. B) Representative flow cytometric analysis images and C) quantification of tumor MDSCs (CD11b^+^Gr‐1^+^) gating on CD45^+^ cells (n = 4). D) Flow cytometry analysis of H_2_DCF MFI in MDSCs from various study groups (n = 4). E) Representative flow cytometric analysis of tumor CD8^+^ and CD4^+^ T cells gating on CD3^+^ cells, and F,G) their statistical data (n = 4). H) Quantitative analysis of CD8^+^IFN‐γ^+^ tumor T cells by flow cytometry (n = 4). I) Western blot analysis of A2AR expression levels in whole‐cell extracts from 4T1 tumor tissues. The gels were run in parallel. J) Characterization of DC maturation in lymph nodes by staining CD86, CD40, and MHCII antibodies (gating on CD11c^+^, n = 4). K) IFN‐γ and L) TNF‐*α* serum levels were detected using ELISA (n = 4). The proportion of M) CD8^+^ T cells gating on CD3^+^, N) CD4^+^ T cells gating on CD3^+^, and O) CD11b^+^Gr‐1^+^ MDSCs gating on CD45^+^ from the spleens of each group (n = 4). P) MDSC ROS content in each group (n = 4). Data are expressed as the means ± SD. Statistical significance is defined as *p < 0.05, **p < 0.01, ***p < 0.001, and ****p < 0.0001 compared with the PPDAIn PTT group, and ns represents no significant difference.

We investigated immune responses upon different treatments. We first assessed whether the combination of A2AR nanoinhibitor delivery with PTT‐induced ICD could relieve the immunosuppressive TME. MDSCs and Tregs are the primary immunosuppressive cells in the TME. Their development closely correlates with adenosine levels and corresponds with poor prognosis by facilitating tumor‐mediated immune escape.^[^ ^32^ ^]^ After different treatments, tumors in each group were harvested and analyzed (**Figure** **7**A). Representative MDSC dot plots (CD11b^+^Gr‐1^+^) showed changes in the proportion of MDSC infiltration after various treatments (Figure 7B). The PPDAIn PTT group had a remarkably reduced tumor MDSC frequency that was 5.38 times lower than that of the PBS group (Figure 7C). The MDSC percentage was also lower in the PPDAIn PTT group compared to the P‐PDAIn PTT group, suggesting that PPDAIn PTT treatment evoked a better immune response, likely due to better accumulation in the tumor. The PPDA PTT group results demonstrated that only PTT treatment did not elicit the intended immune effect. We further investigated the MDSC function in each group by staining with 2′,7′‐Dichlorofluorescein diacetate (H_2_DCF) to measure the intracellular reactive oxygen species (ROS) content.^[^ ^9^ ^]^ There was a remarkable decrease in H_2_DCF MFI in the PPDAIn PTT group (Figure 7D), indicating that PPDAIn PTT treatment decreased the ability of MDSCs to generate ROS, thus impairing its inhibitory effect on lymphocytes. Additionally, we characterized tumor Tregs using immunohistochemistry (IHC). Consistently, the PPDAIn PTT group also significantly decreased the tumor Treg proportion compared with the PBS group (Figure S21, Supporting Information). Moreover, immunofluorescence (IF) staining showed M2 (CD206^+^) macrophage infiltration markedly decreased in PPDAIn PTT group, while M1(iNOS^+^) increased (Figure S22, Supporting Information).

Extracellular adenosine has been reported to disable CD8^+^ T cell cytotoxic effector function, which contributes to tumor cell immune evasion.^[^ ^33^ ^]^ To explore whether the tumor‐specific adhesive A2AR nanoinhibitor influenced the population and function of CD8^+^ T cells after PTT‐induced ICD, we analyzed tumor tissues collected from each group. The representative CD8^+^ and CD4^+^ T cell dot plots are shown in Figure 7E. Compared with the PPDAIn, PPDA PTT, and P‐PDAIn PTT groups, the percentage of CD8^+^ T cells in the PPDAIn PTT group was increased by 1.68, 2.65, and 1.21 times, respectively (Figure 7F), indicating that the T cell recruitment caused by PPDAIn PTT treatment is superior to any single treatment mode and the non‐responsive group. IF staining further supported that PPDAIn PTT treatment distinctly increased CD8^+^ T‐cell tumor permeation examined by IF staining (Figure S23, Supporting Information). We next analyzed CD8^+^IFN‐*γ* ^+^ T cells and found that there was a significant increase in tumors treated with PPDAIn PTT compared to P‐PDAIn PTT treated tumors (Figure 7H), as well as a striking 4.65‐fold increase compared to the PBS group. Taken together, these data revealed that PTT combined with efficient A2AR blockade enhanced CD8^+^ T cells in 4T1‐bearing mice.

Additionally, we found that expression of MMP9, a marker positively associated with tumor metastasis, was significantly reduced in the PPDAIn PTT treated group compared with the PBS group (Figure S24, Supporting Information). Ki67 expression was also markedly reduced in tumor tissue after P‐PDAIn PTT and PPDAIn PTT treatments, as assessed by IHC. However, it is worth noting that Ki67 expression, which is related to malignant tumor cell proliferation,^[^ ^34^ ^]^ was higher in the PTT alone group (G3) than in the PBS group, suggesting that photothermal treatment promoted residual tumor cell proliferation (Figure S25, Supporting Information).

Tumor tissue IHC showed that the group treated with PTT alone (without A2AR inhibitor) had remarkably upregulated A2AR expression. The results are likely due to the stimulation of secreted inflammatory mediators during PTT, such as IL‐1*β*.^[^ ^35^ ^]^ Notably, SCH58261 contained group prevented PTT‐induced A2AR upregulation (Figure S26, Supporting Information). The results were further validated by Western blot. The PPDAIn treated groups markedly reduced A2AR protein levels in lysed 4T1 tumors (Figure 7I). Densitometric band quantification was performed to analyze the Western blot results (Figure S27, Supporting Information). The phenomenon of PTT‐promoted A2AR expression suggests that tumor cells have a cunning ability to respond to environmental stimuli and escape antitumor effects via negative feedback from the adenosine‐A2AR metabolic pathway, highlighting the significance of a targeting strategy that blocks this metabolic checkpoint.

We explored the impact of combining the tailored A2AR nanoinhibitor with PTT‐induced ICD on the systemic immune response. DCs are essential APCs and play a pivotal role in the adaptive immune response by effective antigen presentation and T cell activation. Therefore, we used flow cytometry to analyze mature DCs in tumor‐draining lymph nodes (TDLN) after staining with antibodies against costimulatory markers and MHCII. An increase in CD86, CD40, and MHCII expression levels was observed in the PTT group, indicating that DAMP exposure promoted DC maturation (Figure 7J). However, in contrast to the PPDA PTT group, the P‐PDAIn PTT and PPDAIn PTT groups showed a greater increase in DC maturity due to less suppression during ICD and elimination of immunosuppressive cells in the TME (Figure 7J). Consistent with facilitated DC maturation, plasma TNF‐*α* and IFN‐*γ* levels were significantly upregulated in the P‐PDAIn PTT and PPDAIn PTT groups (Figure 7K,L). TNF‐*α* and IFN‐*γ* are critical cytokines released by immune cells which can trigger T‐cell responses. Furthermore, analysis of spleen lymphocyte distribution showed that the number of CD8^+^ T cells was significantly upregulated in the PPDAIn PPT group compared with the control group, indicative of systemic immunity mobilization (Figure 7M). The number of CD4^+^ T cells was decreased in the PPDAIn PPT group (Figure 7N). PPDAIn PPT treatment also led to a significant decrease in MDSC distribution and intracellular ROS content in the spleen compared with the control group (Figure 7O,P), demonstrating that treatment with PPDAIn PPT could not only regulate intratumoral MDSC distribution but also play a crucial role in spleen MDSC regulation. These results demonstrated that the proposed combination strategy could simultaneously reverse the immunosuppressive TME and trigger robust systemic antitumor immunity to inhibit tumor survival synergistically.

## 3 Conclusion

Here we demonstrated that an increase in the immunosuppressive metabolite adenosine acted as a negative feedback regulator during PTT‐induced ICD, severely suppressing antitumor immunotherapy effects. By delivering an A2AR inhibitor to the tumor via PPDA, an adhesive nanocarrier with photothermal properties, we directly targeted the negative feedback generated from the adenosine‐A2AR metabolic pathway during the ICD process to alter the immunosuppressive TME. The nanosystem achieved robust antitumor immune efficacy, with inhibition of both primary and abscopal tumors and reduced tumor metastasis. Targeting the adenosine‐A2AR metabolic pathway could also be applied to other therapeutic mechanisms that induce tumor ICD. Moreover, the strategy presented here may find a wide application in the field of immune metabolism, particularly in overcoming tumor immune evasion by immune response modulation.

## 4 Experimental Section

### Materials

Dopamine, SCH58261, Collagenase A, 2′,7′‐Dichlorofluorescein diacetate (H2DCF), and Coformycin (Erythro‑9‑(2‑hydroxy‑3‑nonyl) adenine) were purchased from Sigma‐Aldrich. 4‐bromomethyl phenylboronic acid (BPBA), Amino polyethylene glycol (PEG), Rhodamine B isothiocyanate (Rhodamine B), and Fluorescein5(6)‐isothiocyanate (FITC) were acquired from Aladdin Industrial Corporation. Bodipy and Live/Dead staining kits were obtained from Thermo Fisher Scientific. Adenosine, CCK‐8, FITC‐Annexin V Apoptosis Detection Kit, Enhanced ATP Assay Kit, BCA assay kit, and Protease inhibitor cocktail for general use (100X) were purchased from Beyotime Biotechnology. Percp‐CRT antibody was purchased from StressMarq Biosciences Inc. Antibodies of APC‐CD73, Alexa Fluor 488‐HMGB1, CD16/32, Foxp3, APC‐CD11c, FITC‐CD80, PE‐CD86, PE‐CD40, FITC‐MHCII, FITC‐CCR7, PE‐CD45, PEcy7‐Gr‐1, APC‐CD11b, APC‐CD3, FITC‐CD4, PEcy7‐CD8*α*, and PE‐IFN*γ* were purchased from BioLegend. The antibody of A2AR was obtained from Proteintech Group, Inc. Adenosine ELISA Kit was obtained from Shanghai Jianglai Biological Technology Co., Ltd. ELISA kits of TNF‐*α*, IL‐6, IFN‐*γ*, and IL‐12p40 were purchased from Dakewe Biotech Co., Ltd. Cytokines of GM‐CSF, IL‐4, and IL‐2 were purchased from Shanghai Jinan Technology Co., Ltd. DNase I and Bouin's solution were purchased from Beijing solarbio science&technology co., ltd.

### Preparation of Polydopamine Nanocarrier (PDA)

PDA was prepared according to the previously reported method.^[^ ^25^ ^]^ Briefly, deionized water (45 mL) was added into a clean glass beaker, and then pure ethanol (20 mL) and aqueous ammonia solution (1.5 mL, 28–30%) were added under mild stirring in 30 min. Subsequently, dopamine hydrochloride (0.25 g) dissolved in deionized water (5 mL) was added to the above solution drop by drop. The reaction lasted for 24 h at room temperature. The resultant PDA dispersion was then dialyzed against deionized water for 72 h.

### Synthesis of PBA‐PEG

4‐Bromomethyl phenylboronic acid (BPBA, 45 mg) and amino polyethylene glycol (PEG, 0.35 g) were dissolved with dimethyl sulfoxide (DMSO) in a clean flask. 24 µL triethylamine was slowly added into the mixture under stirring. The reaction was allowed to proceed for 30 h at 70 °C. The obtained product was purified by a two‐step dialysis process, first with DMSO for 48 h, then with deionized water for another 48 h.

### Preparation of PPDA

PBA‐PEG was introduced into PDA by reacting phenylboronic acid groups in PBA‐PEG with catechol groups in PDA. PPDA was simply prepared by mixing PBA‐PEG and PDA at a mass ratio of 4:1 in Tris‐HCl buffer (30 mm, pH = 8.5) under stirring for 24 h. PPDA was subsequently collected by centrifugation at 14 000 rpm and washed with deionized water three times.

To determine the amount of PBA‐PEG modified on PDA, the obtained PPDA NPs were suspended in acid PBS (pH 6.0) to detach the PBA‐PEG segment. The PBA‐PEG in the supernatant after centrifugation was determined by UV–vis absorption analysis.

### Characterization of PDA Photothermal Performance

Purified PDA NPs were quantified by UV–vis absorption spectra or freeze‐drying and weighing. Then PDA dispersed with water into different concentrations (25, 50, 100, and 200 µg mL^−1^). 400 µL of PDA suspension solution at a specific concentration was irradiated with an 808 nm laser for 10 min at a power of 0.50 W cm^−2^. Next, the effect of power density on temperature was investigated at 0.25, 0.50, 0.75, and 1.00 W cm^−2^ with the PDA concentration of 100 µg mL^−1^. Temperature changes were detected by a thermal camera (DALI TECHNOLOGY, LT3‐P) and recorded every ten seconds. Infrared thermal images were further acquired every minute. PDA of 200 µg mL^−1^ was irradiated with 0.5 W cm^−2^ for five consecutive photothermal and natural cooling cycles to examine photothermal stability.

### Photothermal Conversion Efficiency Calculation

PDA or PPDA NPs (200 µg mL^−1^) was exposed to 808 nm irradiation at 0.5 W cm^−2^ for 10 min, and then the solution was cooled down to room temperature. The temperature of the solution was recorded at an interval of 10 s. The photothermal conversion efficiencies (*η*) were measured as follows:^[^ ^25^ ^]^

(1) $$\eta =\left[\mathrm{hs}\;\;\left(T_{\mathrm{Max}}-T_{\mathrm{Surr}}\right)\;-\;Q_{\mathrm{Dis}}\right]/\left[I\;(1-10^{-A808})\right]$$

Where *h* represents the heat transfer coefficient; *s* is the surface area of the container; *T*_max_ and *T*_Surr_ are the steady‐state maximum temperature and the environmental temperature, respectively; *Q*_Dis_ is the heat dissipated from the laser mediated by the solvent and container; *I* represent the laser power, and *A* represents the absorbance at 808 nm.

(2) $$\mathrm{hs}=mC_{\mathrm{water}}/\tau_{s}$$

Where *m* is the mass of the solution containing the photoactive material, *C*_water_ is the specific heat capacity of the water (4.2 J/(g °C)), and *τ*_s_ is the associated time constant, which can be calculated as follows during cooling process.

(3) $$t=-\tau_{S}\;In\;\left(\theta \right)$$

(4) $$\theta =\left(T-T_{\mathrm{Surr}}\right)/\left(T_{\mathrm{Max}}-T_{\mathrm{Surr}}\right)$$

### Characterization of the PDA and PPDA

PDA and PPDA were photographed by TEM (JEM‐1230) and SEM (S‐4800, Japan) for morphology observation. The size and zeta potential of PDA and PPDA were measured by DLS (Malvern Instruments Ltd., Worcestershire, UK). The size variations of PDA and PPDA in PBS (pH = 7.4 and 6.2) were further monitored by DLS. PEG, BPBA, PEG‐PBA, PPDA, PDA, and aPDA were lyophilized and then analyzed by FTIR. PEG, BPBA, PEG‐PBA, PDA, and PPDA were dispersed in D_2_O for the ^1^H NMR spectrum test (AVANCCEIII600MHz, Bruker).

### Evaluation of Nanoparticle PA Performance

A PA imaging device was utilized to assess the photoacoustic properties of PDA. PDA solution was injected into the hole of agarose gel molds for analysis. PA signals were acquired under the laser wavelength of 750 nm. For in vivo PA imaging, tumor‐bearing BALB/c mice were anesthetized with isoflurane, and PA images were acquired with the laser wavelength 750 nm before NPs injection. Then PDA, P‐PDA, or PPDA suspension (25 mg kg^−1^) was intravenously injected, and the PA images were obtained at 2, 4, 6, 10, and 24 h after injection. All animal experiments were carried out according to the Tongji Ethics Committee for Animal Experiments guidelines (TJAA07720102).

### Drug Loading Ability of PPDA

SCH58261 was dispersed in DMSO and then added dropwise to PPDA suspension under vigorous stirring. The suspension was stirred for 24 h. PPDAIn was acquired by centrifugation (14 000 rpm, 15 min) to remove the unloaded SCH58261. The unloaded inhibitor amount was determined by the absorbance of the supernatant at 283 nm, and the drug‐loading encapsulation efficacy was calculated following the formula: Encapsulation efficacy (%) = (Loaded drug/ Total drug added) × 100%.

### Drug Release

To assess SCH58261 release kinetics with the stimulation of pH and light irradiation, 0.125 mL of PPDAIn were packaged in a dialysis bag (MWCO = 1.0 kDa) and then immersed within 25 mL of PBS buffer. The pH value of PBS buffer was tuned from 7.4 to 6.2 for the assessment of pH‐responsive release. For irradiation‐stimulated release, PPDAIn samples were irradiated for 30 min at time points of 4 and 6 h. PBS solution at different time points was analyzed by UV–vis spectrometer to determine the concentration of released SCH58261.

### Cell Culture and Cytotoxicity of Nanoparticles

4T1 was obtained from the Chinese Academy of Sciences cell library, cultured in Dulbecco's Modified Eagle Medium (DMEM, Hyclone) supplemented with 10% FBS (Lonsera), 100 U mL^−1^ penicillin, 0.1 mg mL^−1^ streptomycin (complete culture media) in a cell incubator (37 °C with 5% CO_2_). CCK‐8 assay was used to determine the cytotoxicity of PPDA in complete culture media. Briefly, 4T1 cells were seeded into 96‐well plates (150 µL, 1 × 10^4^ cells/ well). After 12 h incubation, the medium was removed and changed to fresh mediums containing different concentrations of PPDA. 4T1 cells were subsequently incubated for another 24 h. Then, the mediums with PPDA were removed and washed with PBS three times. A fresh medium containing 10% CCK‐8 was added to each well and incubated for 2 h in an incubator. The absorbance values were measured at 450 nm by a microplate reader (Thermo Fisher Scientific, USA).

### The Interaction of PPDA with Tumor Cells

1.5 mg FITC was dissolved in 100 µL DMSO and was added dropwise to the PPDA dispersed with 0.05 m NaHCO_3._ The reaction was allowed for 24 h. Subsequently, the dispersion was centrifuged to remove unconjugated FITC. A part of FITC‐PPDA was dispersed in PBS (pH 6.2, 10 mm) to shed the PEG crown. After 6 h incubation, the above suspension was centrifuged and washed three times with water to obtain FITC‐aPDA. The preparation method of Rhodamine B‐PPDA and rhodamine B‐aPDA was the same as the above conditions. Fluorescein‐labeled NPs in a quartz cuvette were measured to obtain its Fluorescence spectra.

4T1 cells were seeded into confocal dishes (Nest, 35 mm) at a density of 2.0 × 10^5^ cells per dish. After incubation for 12 h, FITC‐PPDA or FITC‐aPDA dispersed in complete DMEM (100 µg mL^−1^) was added into the dishes incubated for 1 or 2 h. Then, the treated cells were fixed with 4% paraformaldehyde for 10 min and rinsed three times with PBS. Subsequently, the cells were covered with an antifade mounting medium containing Hoechst 33342. Next, samples were examined using a CLSM (Nikon A1R, Japan). 4T1 cells treated with FITC‐PPDA or FITC‐aPDA were also collected and detected by flow cytometry (FACSVerse, BD), and data were analyzed using FlowJo 7.6.1.

Next, the interaction of nanocarrier with tumor cells was evaluated using a multicellular spheroid tumor model. 4T1 cells were diluted in a mixture of 1.2% methylcellulose/ DMEM medium (1/4, v/v) with a density of 1 × 10^6^ cells mL^−1^. Cell suspension of 20 µL per sample was dropped at the lid of the cell‐culture dish. Then, the lid was gently turned over and placed onto a dish containing PBS solution. Subsequently, the dish with cell samples was incubated in an incubator for three days. Next, tumor spheroids were removed into 1.0% (w/v) agarose pre‐coated 24‐well plate and treated with Rhodamine B‐PPDA or Rhodamine B‐aPDA (100 µg mL^−1^) for 12 h, respectively (n = 3). CLSM Z‐stack scanning (Leica SP8) was utilized to monitor the accumulation capability of different NPs in tumor spheroid. Image J software was further used to quantify the fluorescence intensity.

### Preparation of P‐PDA

The synthesis of PDA with a non‐responsive PEG shell (P‐PDA) was according to the previously reported method.^[^ ^36^ ^]^ Briefly, PDA was dispersed in alkaline buffer solution (pH = 9), then NH_2_‐PEG was added at a mass ratio of 1:3. After vigorously stirring for 24 h, the product was purified by centrifugation and washed with deionized water three times.

### Biodistribution of Nanoparticles

PDA was labeled with Bodipy 650/665‐X NHS Ester. Then, the Bodipy‐PDA was further used to prepare Bodipy‐PPDA and Bodipy‐P‐PDA. 4T1 tumor‐bearing BALB/c mice were injected with 150 µL of different NP suspensions (25 mg kg^−1^), respectively (n = 3). The mice were euthanized at 24 h post‐injection. The major organs and tumors were collected and imaged by a living animal imaging system (Ex/Em = 630/680 nm, AniView 100, China). MFI of the region of interest (ROI) was calculated by the software supporting the system.

### Photothermal Cytotoxicity

4T1 Cells were seeded in a 24‐well plate for 12 h. Then the 4T1 cells were treated with fresh complete DMEM containing PPDA (100 µg mL^−1^) for 4 h. Cells were treated with an 808 nm laser for 10 min and controlled at a specific temperature (42, 45, 48, and 51 °C). After 12 h, the cells were collected and stained with FITC‐Annexin V Apoptosis Detection Kit according to the manufacturer's instructions. Briefly, collected all cells, including supernatant and adherent cells. Then, they were stained with Annexin V‐FITC (5 µL for one sample) and PI (10 µL for one sample) for 20 min at room temperature in the dark and then immediately analyzed by flow cytometry (FACSVerse, BD).

4T1 cells were seeded into confocal dishes at 2 × 10^5^ cells per dish and attached for 12 h. The cells were then treated with PPDA (100 µg mL^−1^) for 4 h. Then, cell samples were irradiated with an 808 nm laser for 10 min and incubated with a fresh medium. Next, all the samples were incubated for another 4 h and stained with Calcein/ PI (Live/Dead staining kit) for 15 min according to the manufacturer's instructions. Finally, samples were imaged with a fluorescence microscope (Leica SP8, Germany).

### Detection of CRT

4T1 cells were cultured in 24‐well plates in 1 mL (1 × 10^5^ cells/ well) for 24 h and then incubated with PPDA (100 µg mL^−1^) for 4 h. Afterward, PTT groups were irradiated using an 808 nm laser for 10 min and washed with PBS. After a further incubation of 12 h, cells were fixed with 1% paraformaldehyde solution for 20 min. After that, the cells were blocked with 5% FBS in PBS solution for 30 min. Then, the Percp‐CRT antibody was diluted by 1:100 and incubated with the cell samples for 1 h at room temperature. Finally, flow cytometry was used to do quantitative research. CLSM analysis was also implemented after staining with Hoechst 33342.

### Detection of HMGB1

4T1 cells were seeded in 24‐well plates. PTT groups were irradiated using an 808 nm laser for 10 min, and the temperatures were controlled at 42, 45, 48, and 51 °C, respectively. After being cultured for 12 h, cell samples were washed, fixed, and permeabilized by 0.5% Triton X‐100 in PBS for 10 min at room temperature. After thorough cleaning three times, samples were blocked for 30 min. Then, the antibody of Alexa Fluor 488‐HMGB1 was incubated with cell samples for 3 h at room temperature. Subsequently, samples were washed three times with washing buffer solution and then covered by an antifade mounting medium with Hoechst 33342 for CLSM observation. Co‐localization analysis was used by LAS X software.

### Detection of ATP

The extracellularly released ATP was examined with an Enhanced ATP Assay Kit. 4T1 cells were cultured and irradiated by the above method. The released ATP in the cell supernatant was detected at 1, 2, 4, 6, 8, and 12 h according to the manufacturer's protocol.

### Detection of Adenosine In Vitro

4T1 cells in 24‐well plates were irradiated for 10 min controlling at 48 °C by adjusting power density. According to the manufacturer's protocol, the cell supernatants were collected at 2, 4, 8, and 12 h and detected by Adenosine ELISA Kit.

### Detection of Adenosine In Vivo

4T1 tumor‐bearing mice were irradiated at designed time points. Mice were euthanized, and tumors were removed, then rinsed with cold PBS and weighed. Normal mammary glands were also collected from non‐tumor‐bearing mice. After tumor tissues were cut into pieces, they were separately mixed with their corresponding volume of PBS (weight to volume ratio of 1:4) on ice and thoroughly ground. The PBS contained a Protease inhibitor cocktail (1 Х) and Erythro‑9‑(2‑hydroxy‑3‑nonyl) adenine (10 µm) to prevent adenosine degradation. Finally, tissue homogenates were centrifuged at 5000 × g for 10 min, and the supernatant was taken for ELISA testing according to the manufacturer's instructions.

For high‑performance liquid chromatography (HPLC) detection, the supernatants of tissue homogenates were freeze‑dried and re‑dissolved by deionized water of 1/10 of the initial volume. Then, the samples were filtered with a 3000 Da cut‑off filter. Adenosine measurement was then performed using HPLC (mobile phases: acetonitrile and 10 mm KH_2_PO_4_, v/v of 1/9. Flow rate: 1 mL min^−1^. Detection wavelength: 260 nm).

### Detection of the Expression of CD73

Tumor tissues were excised 48 h after treatments, cut into pieces, and digested into single‑cell suspensions using collagenase (2 mg mL^−1^) and DNase I (0.6 units mL^−1^). Cell suspensions were washed with cold PBS, counted, and taken out one million cells for APC‐CD73 antibody staining. Cell samples were first blocked by anti‐CD16/32 and then stained with the CD73 antibody using the manufacturer's protocol. Finally, samples were monitored by Flow cytometry.

### Detection of cAMP Inhibition

DC, macrophages, or T cells were seeded into 6‐well plates (1 × 10^6^ cells/well), followed by treated under different conditions. G1) PBS, G2) adenosine (40 µm), G3) adenosine plus PPDA (PPDA, 100 µg mL^−1^), G4) adenosine plus nonphotoirradiated PPDAIn (PPDA, 100 µg mL^−1^; SCH58261, 48 µm), G5) adenosine plus photoirradiated PPDAIn (PPDA, 100 µg mL^−1^; SCH58261, 48 µm; NPs irradiated at 48 °C for 30 min), and G6) adenosine plus free SCH58261(48 µm). After incubation for 24h, cells were lysed with Cell lysis buffer (Beyotime Biotechnology). Supernatants were collected by centrifugation for cAMP detection. The measurement was performed using the cAMP detection ELISA kits (Shanghai Jingkang Biological Engineering Co., Ltd.).

### Detection of BMDC Maturation In Vitro

BMDCs were isolated from femurs and tibias of BALB/c mice (7 weeks old). RPMI 1640 complete medium (containing 10% FBS, 100 U mL^−1^ penicillin, and 0.1 mg mL^−1^ streptomycin) supplemented with GM‐CSF (20 ng mL^−1^) and IL‐4 (10 ng mL^−1^) was used to culture BMDCs in 6‐well plates with 4 mL at a cell density of 1 × 10^6^ cells mL^−1^. On days 2 and 4, the culture medium was gently replaced with the above fresh medium. On day 6, immature BMDCs were harvested for further experiments.

On day 6, immature BMDCs were counted and seeded into 24‐well plates at a density of 2 × 10^5^ cells per well at the lower chamber of the Transwell. Tumor cells of 1 × 10^5^ were placed in the upper chamber with different treatments. After 24 h incubation, the BMDCs were washed and stained with antibodies, including anti‐CD16/32, APC‐CD11c, FITC‐CD80, PE‐CD86, PE‐CD40, FITC‐MHC II, and FITC‐CCR7. Finally, all the samples were re‐suspended in a flow buffer (1% FBS in PBS) before being analyzed by flow cytometry. Supernatants of the BMDC culture mediums were collected and analyzed by ELISA kits of TNF‐*α*, IL‐6, and IL‐12p40 according to the manufacturer's instructions.

BMDCs from different groups were stained with Giemsa dye solution for observing morphology. Briefly, BMDCs were fixed and coated on slides, which were stained with Giemsa A for 1 min, and then stained with B for 3 min. Finally, they were rinsed with water and observed under a microscope (BioTek).

### T‐Cell Proliferation Assay

Single‐cell suspensions of the spleen were acquired by gently grinding, filtering, lysing red blood cells, and washing in a bacteria‐free operating environment. The CFSE was dissolved with DMSO and diluted with PBS to obtain a 5 µm working solution. The above single‐cell suspensions were counted and then suspended in the CFSE working solution at a density of 1.5 × 10^6^ cells mL^−1^ incubating for 20 min at 37 °C. Subsequently, a cold complete RPMI 1640 medium was added to terminate the reaction at 4 °C for 5 min. Then, cells were centrifuged at 1500 rpm for 5 min to remove supernatant and washed three times with a fresh medium. Next, complete RPMI 1640 medium supplemented with IL‐2 (10 ng mL^−1^) was used to culture the obtained cells following stimulated with tumor cells with different treatments for three days, then stained with APC‐CD3 antibody and detected by flow cytometry. Supernatant's culture mediums were analyzed by ELISA kits.

### Antitumor Efficacy In Vivo

The female BALB/c (5–6 weeks) were purchased from the Shanghai Laboratory Animal Center (SLAC, Shanghai, China). The breast tumor model was established by subcutaneous injection of 4T1 cells (1 × 10^6^ cells in 100 µL per mouse) into the right second row of breast pads on day ‐6 to obtain the primary tumor. To form a bilateral tumor model, 4T1 cells (1 × 10^6^ cells in 100 µL per mouse) were subcutaneously injected into the left second row of breast pads on day ‐2 to obtain the distant tumor. All animal experiments strictly followed the Ethics Committee of Tongji University (Shanghai, China) and the Guide for the Care and Use of Laboratory Animals published by the US National Institutes of Health. When the primary tumor volumes were ≈100 mm^3^, 4T1 tumor‐bearing mice were randomly divided into six groups (n = 6) and treated with (G1) PBS, (G2) PPDAIn, (G3) PPDA PTT, (G4) PPDA PTT +In, (G5) P‐PDAIn PTT, and (G6) PPDAIn PTT. The injection dosage of PPDAIn, PPDA, and P‐PDAIn was 25 mg kg^−1^ calculated by PDA. The injected loaded SCH58261 or molecular SCH58261 was 1 mg kg^−1^ in the above groups. The primary tumors of G3, G4, G5, and G6 were irradiated with an 808 nm laser controlling at 48 ± 0.5 °C for 10 min. Mice were treated three times every two other days.

In vivo antitumor efficacy on the primary and abscopal tumor. The tumor size and bodyweight of each mouse were recorded every two days by day 19. The following formula was used to calculate tumor volume: Tumor volume (mm^3^) = 0.5 × length × width^2^. The bilateral tumors and main organs were stained with H&E.

To detect the in vivo efficacy of antitumor lung metastasis, 4T1 tumor models were planted like described above. Mice were sacrificed on day 32, and the excised lungs were fixed in Bouin's solution for 12 h, followed by taking images of the lungs. Besides, the tumor metastasis sites were counted under microscopy by analyzing the yellow surface nodules. After counting, lung tissues were washed with 75% alcohol for H&E staining. The other major organs were also observed through H&E staining.

### Antitumor Immune Response In Vivo

First, 4T1 tumor‐bearing model was established. Female BALB/c mice of 5–6 weeks were subcutaneously injected with 4T1 cells (1 × 10^6^ cells in 100 µL per mouse) at the right second row of breast pads. After seven days, the tumor volume was about 100 mm^3^. Mice were randomly divided into six groups and treated as the above section of “Antitumor efficacy in vivo”. On the 11th day, the mice were euthanized.

Tumors were collected and cut into small pieces before incubation with 2.0 mg mL^−1^ collagenase A and 0.2 mg mL^−1^ DNase I for 60 min at 37 °C. The digested tissues were filtered through a 70 µm cell strainer to obtain single‐cell suspensions. Cell samples were washed with PBS and re‐suspended in cell staining buffer. Cells were counted and aliquoted into 1.5 mL vials (1 × 10^6^ cells/ vial) and, first, blocked with anti‐CD16/32 to avoid non‐specific adsorption. Next, cell samples were separately stained with dye‐conjugated antibodies, including PE‐CD45, PEcy7‐Gr‐1, APC‐CD11b, APC‐CD3 FITC‐CD4, PEcy7‐CD8*α*, and PE‐IFN‐*γ*. The cell‐surface staining was performed for 30 min at 4 °C. For intracellular staining, cells were fixed, permeabilized, and washed according to the manufacturer's protocol. The MDSCs were also stained with H2DCF (10 µm). Tumor tissues were also used for IF and IHC analysis for characterizing Treg and CD8^+^ infiltration and the expression of A2AR, MMP9, and Ki67.

Draining lymph nodes were obtained, then gently ground with rubbers of syringes and filtered to acquire single‐cell suspensions. Next, samples were blocked with anti‐CD16/32. Subsequently, Antibodies of APC‐CD11c, PE‐CD80, PE‐CD40, and V450‐MHCII were stained at 4 °C for 30 min.

After ground, filtered, lysed with erythrocyte, and washed, single‐cell suspensions of spleens in different groups were obtained. The percentages of CD3^+^CD4^+^ T cells, CD3^+^CD8^+^ T cells, and CD45^+^CD11b^+^Gr‐1^+^MDSCs were analyzed using flow cytometry.

Mouse peripheral blood was obtained through the orbit. Serum was acquired by centrifugation at 4000 rpm for 15 min. The amounts of cytokines (IFN‐*γ* and TNF‐*α*) were quantified by ELISA (n = 4) according to the manufacturer's instructions.

### Western Blot

Briefly, after different treatments, tumor tissues were collected and were lysed with RIPA lysis buffer (Beyotime, China). The concentration of protein was quantified by the BCA assay kit. Protein was denatured in loading buffer (Beyotime, China) by boiling for 5 min. Then Western blotting was conducted according to standard protocol. Rabbit A2AR antibody (1:1000) was used as primary antibody, GAPDH (1:3000) was used as the loading control. Finally, protein bands were detected by an enhanced chemiluminescence detection kit.

### Statistical Analysis

Analysis of flow cytometry data was performed by FlowJo 7.6.1 software. Data were presented as the mean ± standard deviation (S.D.). Statistical analysis was implemented using an unpaired Student's t‐test or one‐way ANOVA. The significance analysis was assessed using GraphPad Prism (7.0). Asterisks indicate significant differences: *p < 0.05, **p < 0.01, ***p < 0.001, and ****p < 0.0001. *p* values > 0.05 represent non‐significance (ns).

## Conflict of Interest

The authors declare no conflict of interest.

## Supporting information

Supporting Information

## Data Availability

The data that support the findings of this study are available in the supplementary material of this article.
