# Supplementary material for: Targeting the Negative Feedback of Adenosine‐A2AR Metabolic Pathway by a Tailored Nanoinhibitor for Photothermal Immunotherapy
Source: Adv Sci (Weinh). 2022 Mar 20;9(14):2104182. doi: 10.1002/advs.202104182 (PMC9108638; doi:10.1002/advs.202104182)
Supplement: Supplementary file 1 — Supporting Information [file ADVS-9-2104182-s001.pdf]

Supporting Information

Targeting the negative feedback of adenosine-A2AR metabolic pathway by a tailored nanoinhibitor for photothermal immunotherapy

*Yiqiong Liu, Ying Liu, Dailin Xu, Jie Zang, Xiao Zheng, Yuge Zhao, Yan Li, Ruiqing He, Shuangrong Ruan, Haiqing Dong, Jingjing Gu, Yan Yang, Qian Cheng, Yongyong Li**

Y. Liu, Y. Liu, D. Xu, J. Zang, X. Zheng, Y. Zhao, Y. Li, R. He, S. Ruan, H. Dong, J. Gu, Y. Yang, and Prof. Y. Li
Shanghai Skin Disease Hospital
The Institute for Biomedical Engineering & Nano Science
School of Medicine, Tongji University
Shanghai, 200092, China
E-mail: yongyong_li@tongji.edu.cn

Q. Cheng
Institute of acoustics,
School of Physics Science and Engineering, Tongji University,
Shanghai, 200092, China

Supporting Figures

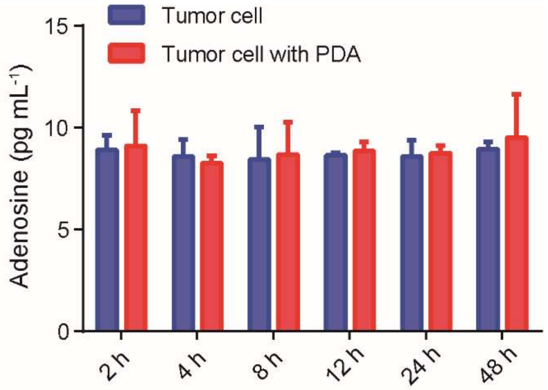

Figure S1. The effect of PDA (100 μg mL^{-1}) on adenosine release in 4T1 cells over time.

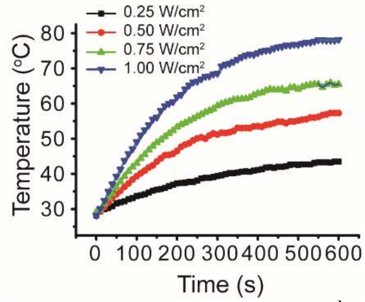

Figure S2. Temperature elevation of PDA NPs (100 μg mL^{-1}) with a near-infrared (NIR) laser at different power densities.

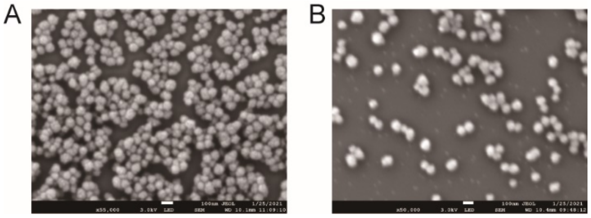

Figure S3. SEM images of PDA (A) and PPDA (B). Scale bars, 100 nm.

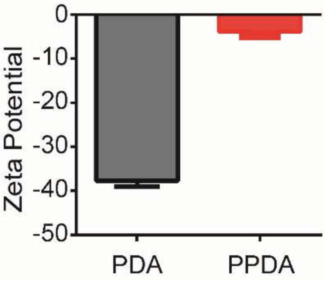

Figure S4. Zeta potential of PDA and PPDA in water (n = 3).

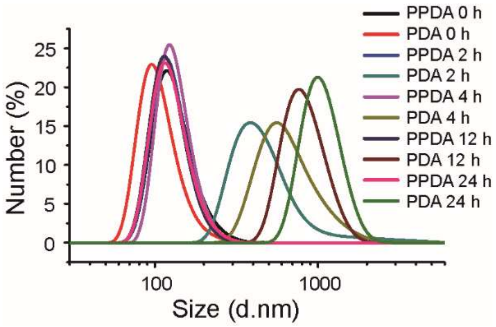

Figure S5. Size stability of PDA and PPDA in PBS (pH = 7.4) over 24 h.

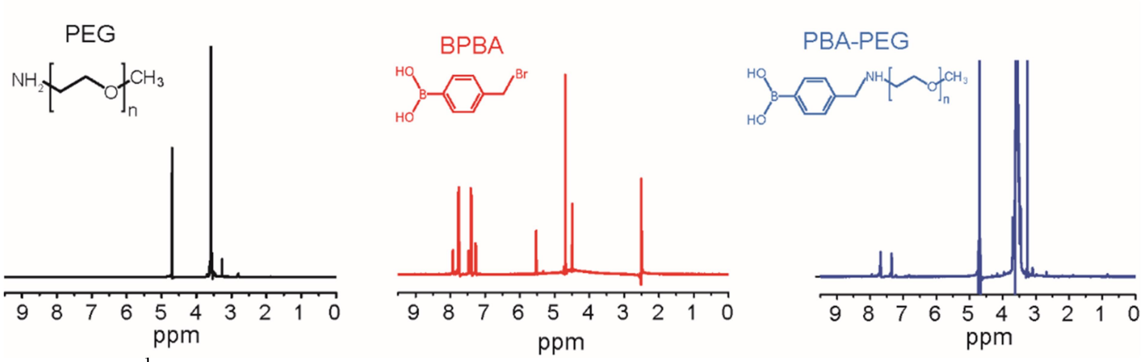

Figure S6. ^{1}H NMR spectrum of PEG, BPBA, and PBA-PEG molecular in D_2O.

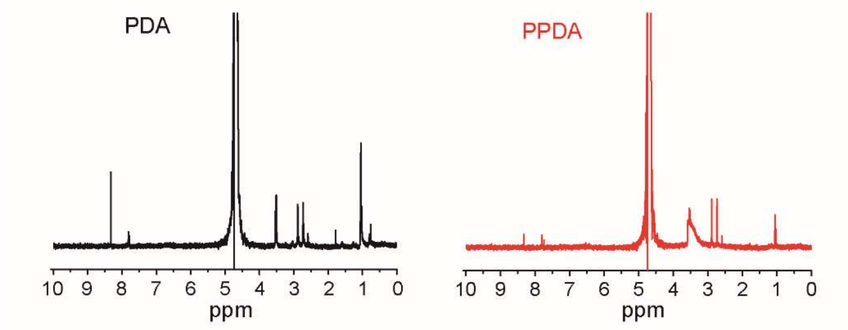

Figure S7. ^{1}H NMR spectrum of PDA and PPDA in D_2O.

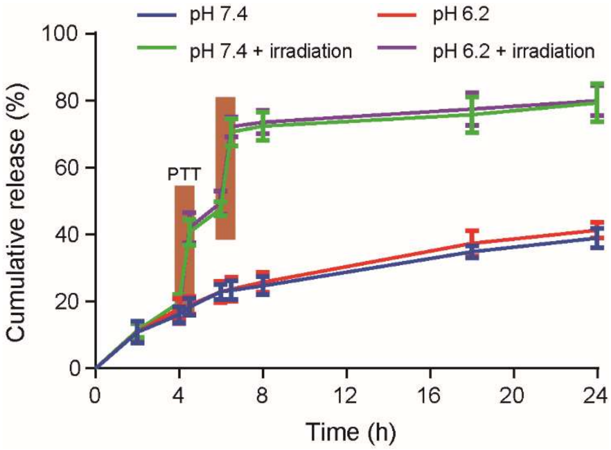

Figure S8. The release profiles of the A2AR inhibitor SCH58261 in normal or acid conditions with or without laser.

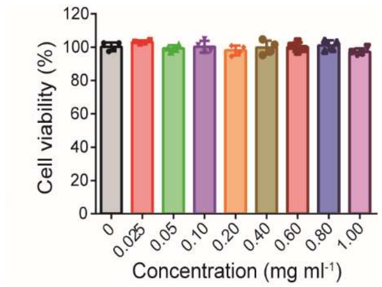

Figure S9. Cell viability of 4T1 cells after incubation with increased concentrations of PPDA NPs.

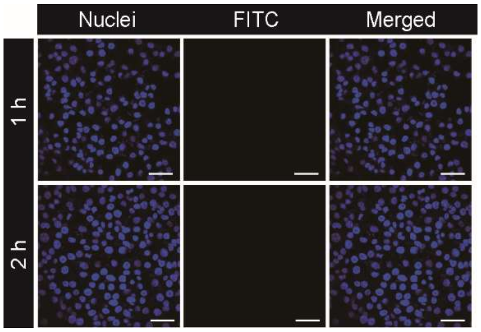

Figure S10. CLSM images of the non-treated 4T1cells (scale bar: 50 μm).

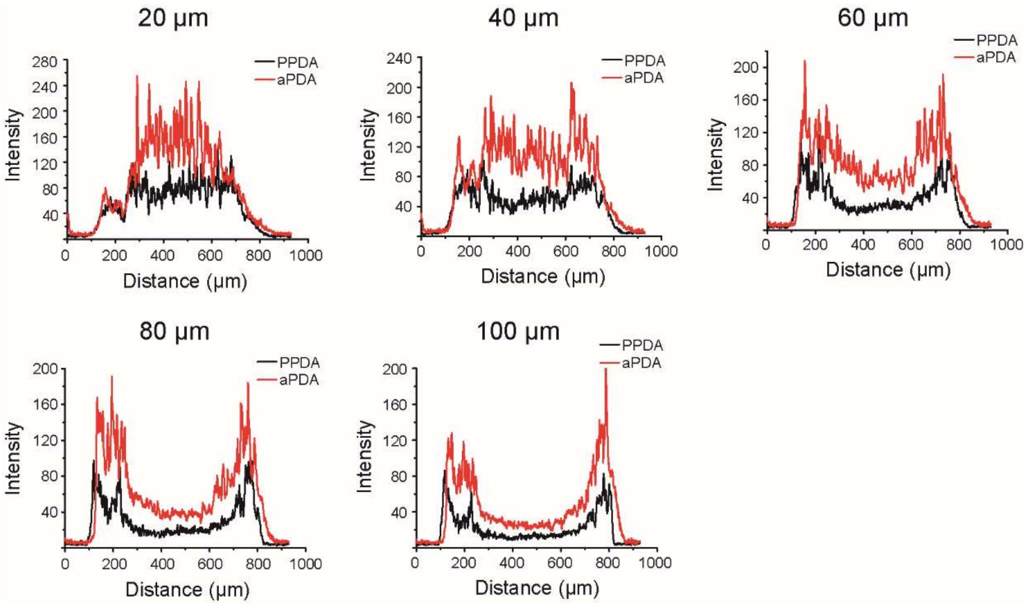

Figure S11. Fluorescence intensity profiles of tumor cell spheroid sections by Z stacking scanning at different depths from the surface (20, 40, 60, 80, and 100 μm, as shown by a white line in Figure 3C).

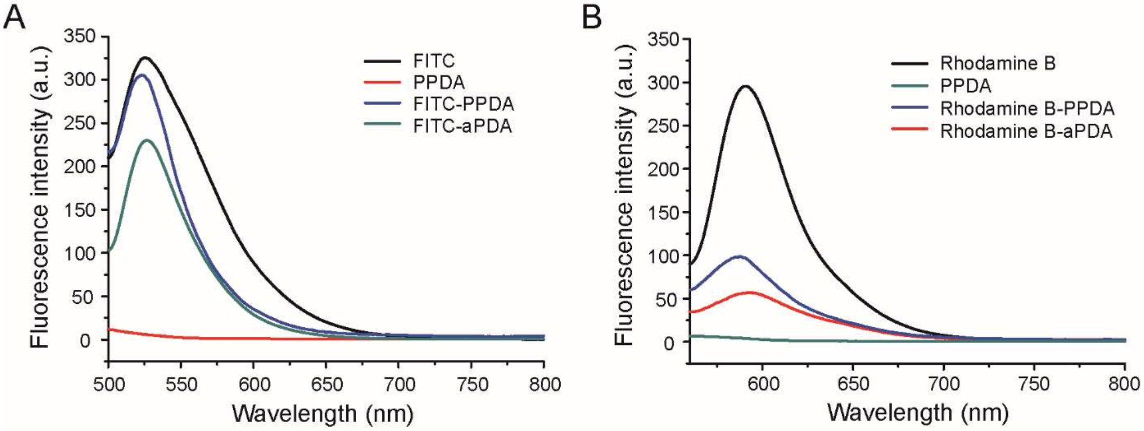

Figure S12. The fluorescence spectra for the FITC(A) and rhodamine-labeled (B) NPs.

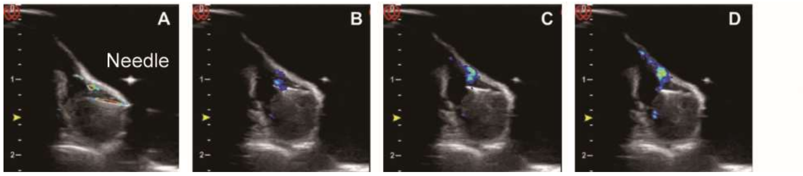

Figure S13. PA detection of PPDA in living mice. Mice were injected intratumorally with PPDA. A) The imaging before injection; B-D) Imaging the injection process.

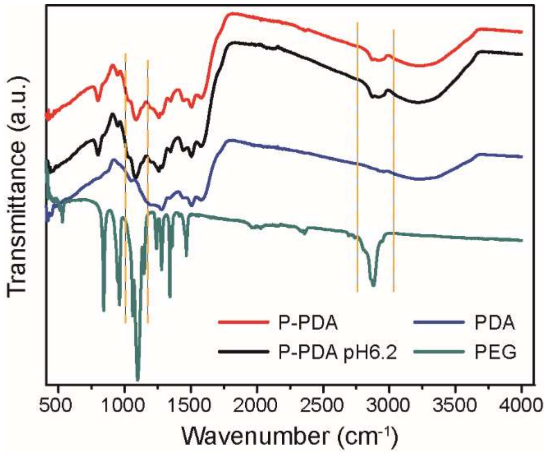

Figure S14. FTIR spectra of PEG, PDA, P-PDA, and P-PDA after acid treatment.

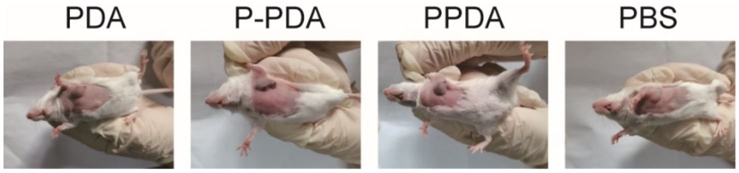

Figure S15. Mice images after injection with different NPs for 6 h. PBS group is the control.

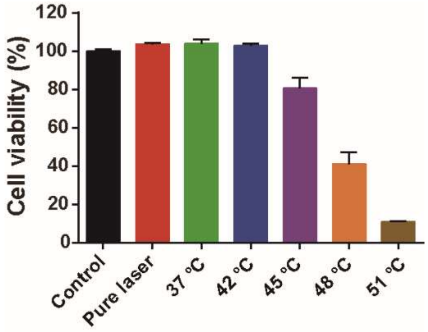

Figure S16. 4T1 cell viability after laser irradiation controlled at different temperatures detected by CCK8 assay.

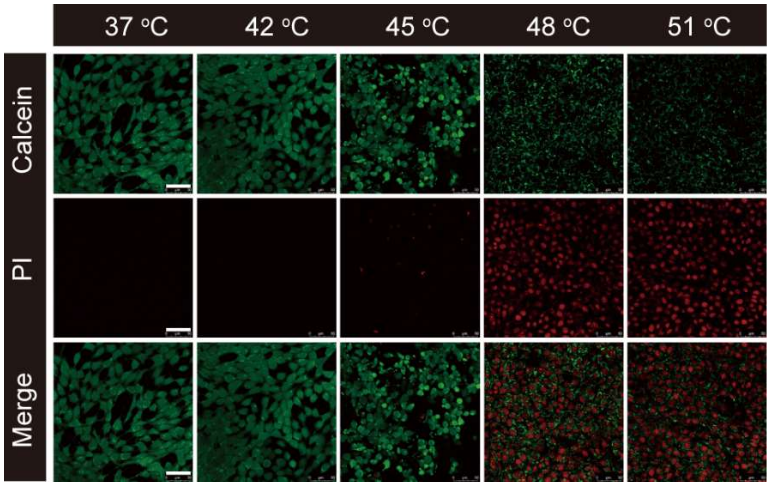

Figure S17. CLSM images of live/dead staining after photothermal treatments for 4 h. Scale bars, 50 μm.

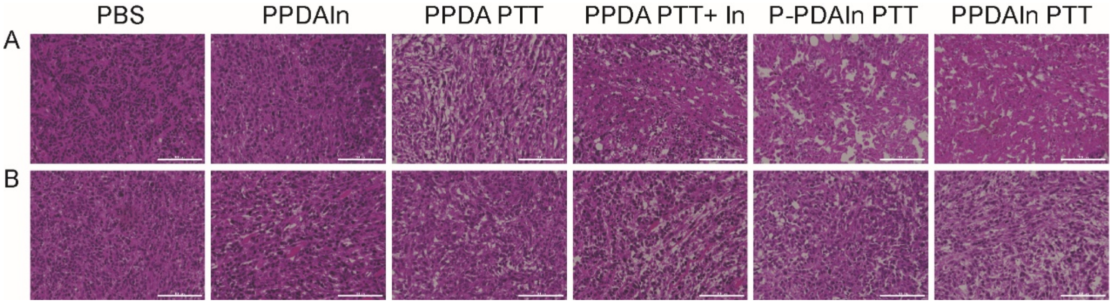

Figure S18. H&E sections of primary (A) and distant tumors (B) with various treatments (scale bars, 100 μm).

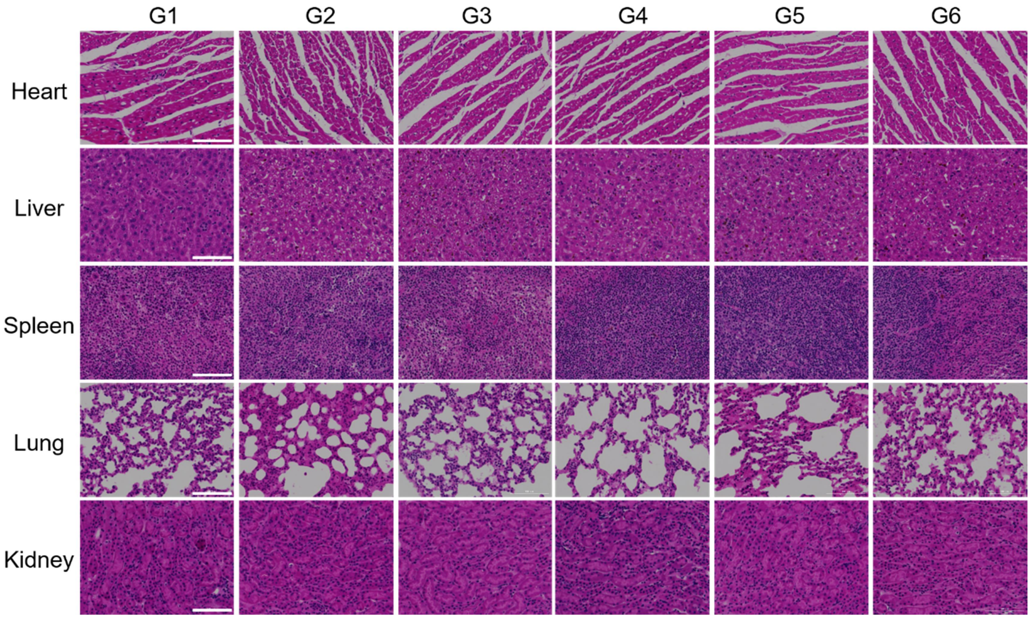

Figure S19. Representative H&E staining of major organs after different treatments. G1: PBS, G2: PPDAIn, G3: PPDA PTT, G4: PPDA PTT+ In, G5: P-PDAIn PTT, G6: PPDAIn PTT. Scale bars, 100 μm.

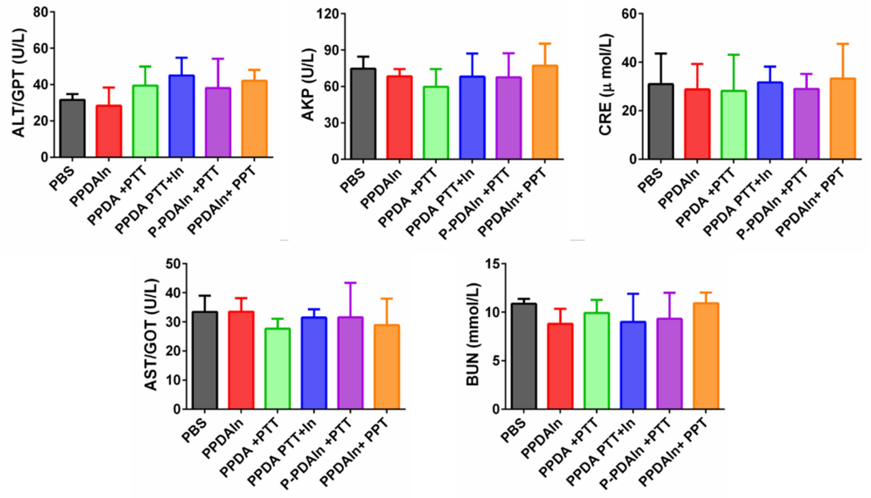

Figure S20. The determination of serum biochemistry parameters including alanine aminotransferase (ALT), alkaline phosphatase (AKP), creatinine (CRE), aspartate aminotransferase (AST), and blood urea nitrogen (BUN).

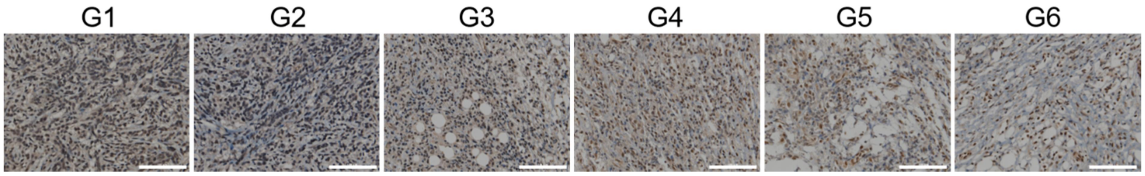

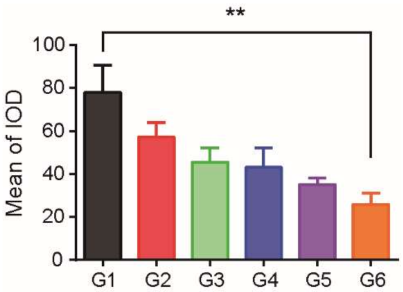

Figure S21. Representative Foxp3 staining images from each group (scale bars, 100 μm), and quantitative analysis of Foxp3 expression by Image-Pro Plus 6.0 (n = 3). IOD indicates integrated optical density. G1: PBS, G2: PPDAIn, G3: PPDA PTT, G4: PPDA PTT+ In, G5: P-PDAIn PTT, G6: PPDAIn PTT.

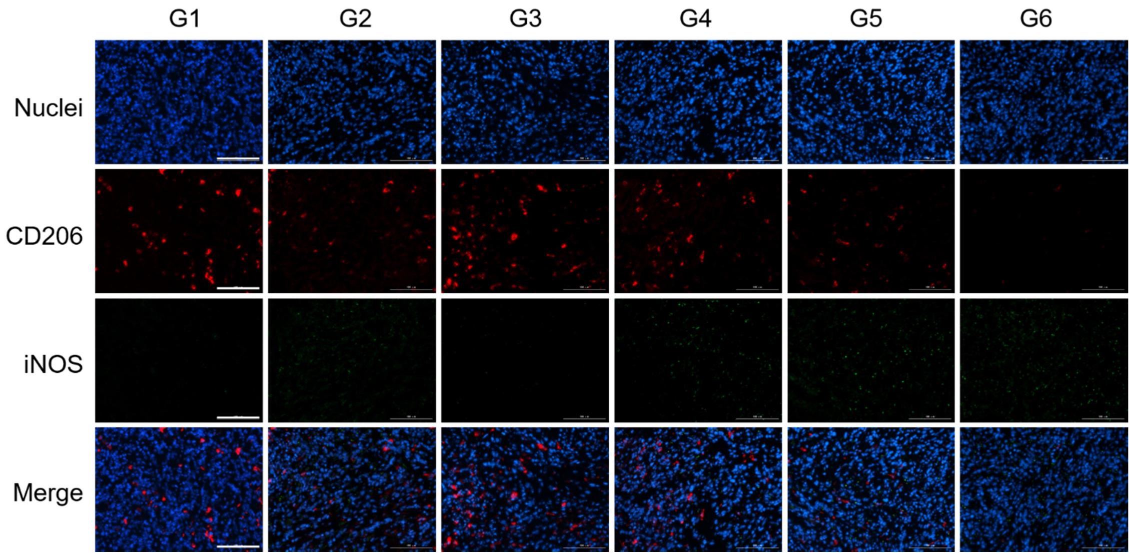

Figure S22. Immunofluorescence staining (green) of the M1 macrophage marker iNOS (green) and the M2 macrophage marker CD206 (red). Scale bars, 100 μm.

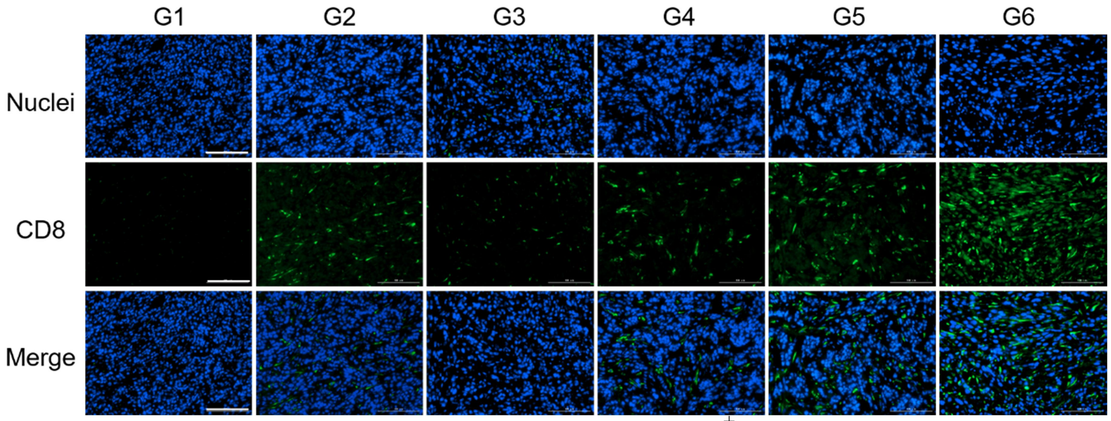

Figure S23. Immunofluorescence images of intratumor CD8^{+} T cells from various groups (green, CD8^{+} T cells; blue, cell nuclei). G1: PBS, G2: PPDAIn, G3: PPDA PTT, G4: PPDA PTT+ In, G5: P-PDAIn PTT, G6: PPDAIn PTT. Scale bars, 100 μm.

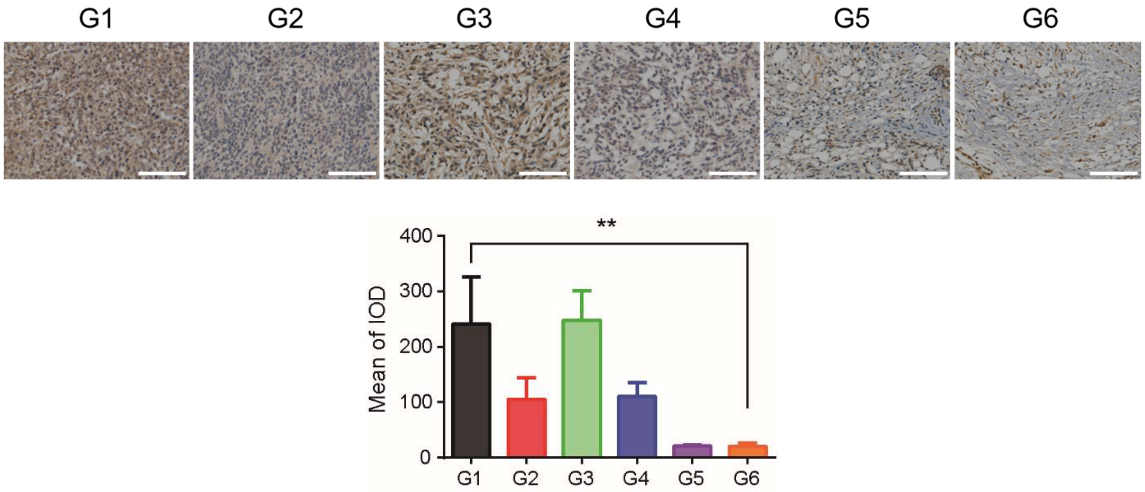

Figure S24. Immunohistochemical analysis of tumors in various groups by labeling with MMP9, and quantitative analysis of MMP9 expression by Image-Pro Plus 6.0 (n = 3). G1: PBS, G2: PPDAIn, G3: PPDA PTT, G4: PPDA PTT+ In, G5: P-PDAIn PTT, G6: PPDAIn PTT. Scale bars, 100 μm.

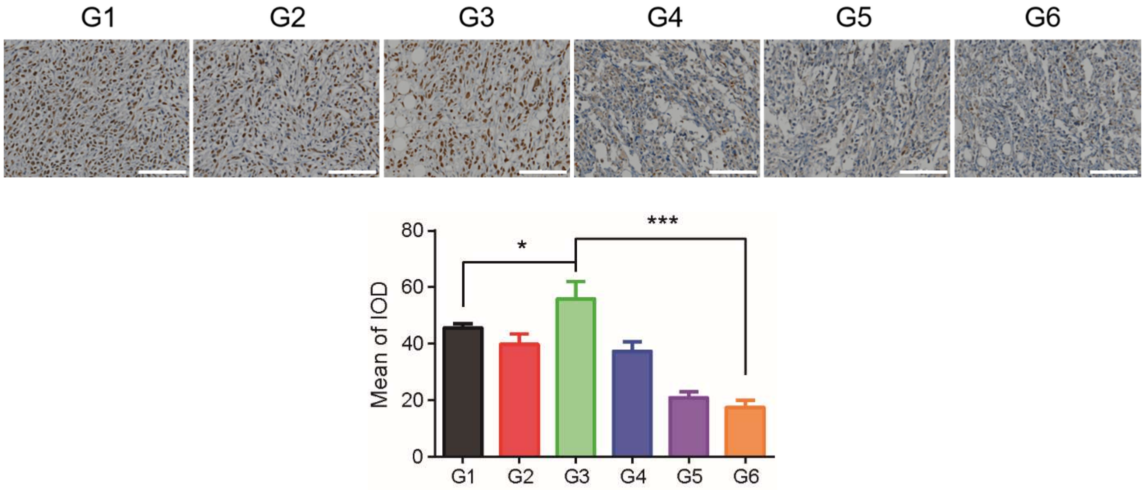

Figure S25. Representative Ki67 staining images from each group (scale bars, 100 μm), and quantitative analysis of Ki67 expression by Image-Pro Plus 6.0 (n = 3). G1: PBS, G2: PPDAIn, G3: PPDA PTT, G4: PPDA PTT+ In, G5: P-PDAIn PTT, G6: PPDAIn PTT.

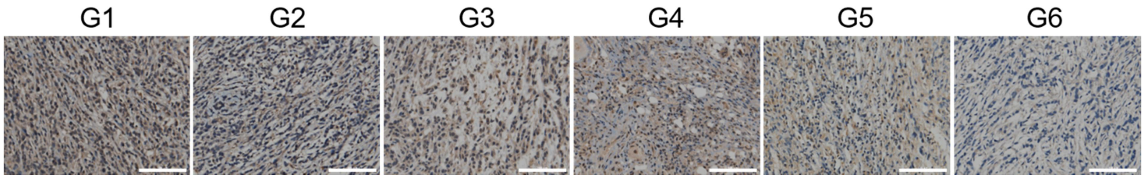

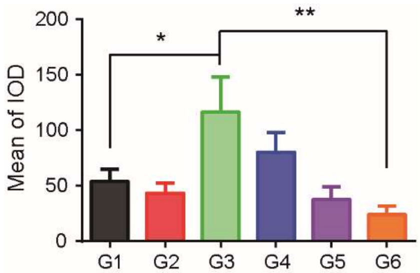

Figure S26. Representative A2AR staining images from each group (scale bars, 100 μm), and quantitative analysis of A2AR expression by Image-Pro Plus 6.0 (n = 3). G1: PBS, G2: PPDAIn, G3: PPDA PTT, G4: PPDA PTT+ In, G5: P-PDAIn PTT, G6: PPDAIn PTT.

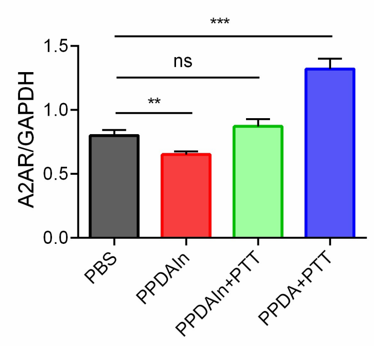

Figure S27. Quantitative statistical analysis of western blots (n=3).
